# Social and emotional skills for nurturing parenting: A qualitative examination

**DOI:** 10.1371/journal.pone.0355716

**Published:** 2026-08-20

**Authors:** Luciana Beccassino, Juliana Borbón, Jorge Cuartas, Catalina Rey-Guerra, Maria Alejandra Gutiérrez-Torres, Melissa Guerra, Helen Baker-Henningham

**Affiliations:** 1 Fundación Apapacho, Bogotá, Colombia; 2 Bangor University, Bangor, United Kingdom; 3 Department of Applied Psychology, New York University, New York, New York, United States of America; 4 Facultad de Economía y Facultad de Ciencias Sociales, Universidad de los Andes, Bogotá, Colombia; 5 Yale University, New Haven, Connecticut, United States of America; 6 University of the West Indies, Mona, Kingston, Jamaica; Touro University California College of Pharmacy, UNITED STATES OF AMERICA

## Abstract

Social and emotional skills develop within cultural contexts and shape parenting behaviors, yet little work has examined the specific skills parents themselves consider important for parenting, or the factors they perceive as supporting these skills, in low- and middle-income countries (LMICs). Centering parents’ own conceptualizations, this two-phase descriptive qualitative study with 54 parents living in Colombia combined an initial phase of focus groups and individual interviews with a second phase of targeted individual interviews, and used thematic analysis to explore the social and emotional skills parents identify as relevant to parenting and the risk and protective factors perceived to hinder or enhance their development. Our findings highlight the importance of emotional awareness, self-regulation, communication, cooperation, and problem-solving skills in adulthood for nurturing parenting and promoting children’s social and emotional development. Parents’ upbringing and support networks emerged as significant factors shaping skill development and child-rearing practices. These findings emphasize the need for policies and interventions, grounded in caregivers’ own perspectives, to promote social and emotional skills for parents in LMIC contexts.

## Introduction

Early childhood is a foundational period of human development marked by heightened neural plasticity and increased sensitivity to environmental influences. This sensitive period lays the foundation for lifelong cognitive, social, emotional, and behavioral trajectories [1]. In these early years, the relationship and interactions between children and their primary caregivers are the main catalysts for skill development [2]. Extensive evidence demonstrates that parenting practices and interactions with children, including engagement in stimulating activities and avoidance of harsh discipline, exert lasting effects on various domains of early childhood development, including social and emotional skills [3,4].

Although parenting practices play a crucial role in early development, interactions between parents and young children vary widely, from highly warm and stimulating engagements to harsh practices such as physical punishment [5]. Some evidence indicates that variations in these parenting behaviors may be partially explained by disparities in parents’ social and emotional skills, including their knowledge, skills, and attitudes to understand and manage emotions, establish supportive relationships, make caring decisions, and achieve personal and collective goals [6]. However, there is limited evidence of the specific social and emotional skills that parents themselves consider relevant for parenting, specifically in low- and middle-income countries (LMICs). These countries are home to 90% of the world’s young children, often face higher levels of contextual adversity (e.g., poverty), and exhibit substantial cultural diversity in the social and emotional skills, parenting behaviors, and child development outcomes that are valued [7].

Evidence on parenting programs show that they can improve parenting practices and children’s cognitive, social, and emotional development [8]. Most of these programs, however, are developed in high-income countries and later transferred to LMIC settings, which has raised concerns about whether their content reflects what parents in these contexts already value, know, and do [9,10]. Scholars increasingly argue that effective and culturally acceptable programs should be grounded in local caregiving values and should engage families from the earliest stages of design and adaptation, rather than importing externally defined models of “good parenting” [11,12]. Approaching parents as active holders of knowledge, rather than as passive recipients of information, is also a matter of epistemic and social justice, given that most of the world’s young children live in contexts that remain underrepresented in developmental research [7,9]. Documenting the social and emotional skills that parents themselves consider relevant to parenting is therefore a necessary first step for designing and adapting interventions that respond to local priorities.

In the present study, we explore the social and emotional skills that a group of parents living in Colombia perceive as relevant to parenting and the contextual factors that act as enablers and/or barriers to developing these skills. Identifying parents’ perspectives of these skills and contextual factors, can inform the design of interventions and ensure interventions are perceived as culturally relevant and acceptable within the context.

### Conceptual framework

In this study, we understand nurturing parenting as a relational dimension of caregiving related to warm, responsive, and emotionally supportive interactions through which parents build secure bonds with their children, respond to their needs, and protect them from harsh or violent treatment. We focus specifically on the parenting skills and interactions that support children’s social and emotional development, and we do not address other domains of care such as health or nutrition. This understanding is consistent with how Colombia’s national technical guidelines for the prevention of violence against young children frame caregiving practices, namely as the relationships that sustain life and, beyond that, give it meaning and protection [13]. Such practices are not limited to meeting basic survival needs, but involve recognizing the individuality of each girl and boy and building meaningful and positive bonds oriented toward development, practices that are built and lived in the everyday life of families and households [13].

This study is grounded in a bioecological systems perspective, which suggests that individuals develop within an environment encompassing interconnected systems, ranging from the closest microsystem, such as family and school, to the broader macrosystem [14]. The proximal processes in the immediate environment, including nurturing parenting, are considered to be the key engines for child development [2]. Numerous studies with samples from various countries and cultures support the importance of these proximal processes in promoting children’s crucial skills such as literacy, numeracy, cognitive, and motor development (e.g., [4,15,16]).

The ecological perspective underscores that parenting behaviors are influenced by a range of factors, including individual characteristics, family dynamics, and cultural contexts. Variations in these broader contextual factors often shape parenting indirectly through proximal influences, such as social and emotional skills, which are both malleable and sensitive to context [17]. Social and emotional skills enable parents to understand and respond to children’s cues, establish strong bonds, foster positive parent-child interactions, model appropriate behaviors, which in turn promote children’s social and emotional development [18,19]. Parents’ social and emotional skills both contribute to, and are reinforced by, lower levels of stress, anxiety, and depression, forming positive feedback loops that promote nurturing parenting [17,20].

Social and emotional skills have been defined through multiple frameworks that offer distinct yet complementary perspectives. Jones et al. [19] propose a model with three interrelated domains: Cognitive regulation (e.g., attention control, working memory), emotional processes (e.g., emotion expression and regulation, self-awareness), and social/interpersonal skills (e.g., prosocial behavior, cooperation, relationship-building). The Collaborative for Academic, Social, and Emotional Learning [6] outlines five core competencies: Self-awareness, self-management, social awareness, relationship skills, and responsible decision-making. Similarly, the Behavioral, Emotional, and Social Skills Inventory (BESSI) [21] categorizes competencies into five domains: Self-management, social engagement, cooperation, emotional resilience, and innovation.

These frameworks provided the conceptual foundation for the development of the measurement instruments and analytical approach used in the current study. We grouped the social and emotional skills reported by parents into three broad, but overlapping, domains for analytic purposes: (1) Emotional skills, including emotion awareness and regulation, as well as recognition and support of children’s emotional experiences, (2) social skills, including communication, trust-building and sharing parenting responsibilities, and (3) adaptability skills, including managing multiple responsibilities, assessing and resolving challenges, and self-efficacy. While we recognize that many of the skills identified by parents, such as self-regulation or learning new skills, cut across these domains, this categorization serves as a heuristic tool to synthesize the themes in a way that remains accessible and grounded in caregivers’ lived experiences. This categorization reflects key priorities and concerns that emerged from our data, including the value of emotional awareness for managing stress, social skills for fostering supportive relationships, and adaptability for coping with adversity. Moreover, this structure provides a meaningful and accessible framework for families, which can inform the design and adaptation of effective interventions.

### Evidence linking social and emotional skills and nurturing parenting

Quantitative research has shown that social and emotional skills are significant predictors of nurturing parenting. Meta-analyses, cross-lagged panel models, and other quantitative studies conducted in Australia, Canada, China, Israel, the U.S., and the U.K. show associations between parents’ emotional awareness, self-regulation, self-efficacy, conflict management, empathy, agency, and social relationships with reduced parenting stress, higher parenting warmth, less hostile behaviors, and improved child social and emotional skills [22–25]. For example, one study found that social and emotional skills like emotional intelligence, assertion, conflict management, empathy, agency, and social skills collectively contributed to 25% of the variance in parenting style, even after controlling for the age and sex of parents and children and child behavior [26].

Echoing results from quantitative studies, some qualitative evidence also highlights the importance of parents’ social and emotional skills for parenting. For example, Bloomfield et al. [27] conducted focus groups with mothers, health visitors, and family support center workers in Hertfordshire, United Kingdom, to explore experiences and perspectives on the challenges and difficulties of parenting. Seventy individuals participated in 12 focus groups, including mothers of children under six from a wide range of socioeconomic and educational backgrounds. While professionals perceived behavioral management as a significant concern for mothers, mothers themselves considered effective communication, understanding their children’s needs, and showing empathy towards their children as their main challenges. Moreover, findings from the focus group highlighted that mothers’ self-efficacy beliefs may be important to their parenting behaviors and their children’s behavior and well-being.

In summary, prior evidence highlights the importance of caregivers’ social and emotional skills for parenting and fostering children’s development. Nonetheless, little is known about parents’ perspectives of the skills they need, especially in LMICs.

### The Colombian context

In LMICs, contextual stressors such as poverty and violence are factors that affect parents’ ability to develop and use social and emotional skills. Constant hardship can elevate levels of stress and anxiety, contributing to higher risk for depressive symptoms, post-traumatic stress (PTS), and general psychological distress [28,29]. These psychological consequences can undermine individuals’ ability to identify and regulate emotions effectively, engage in positive social interactions, accurately interpret social cues, and adapt to shifting situations and demands. A low level of social and emotional skills can in turn compromise responsive and effective parenting, and the provision of a stable and supportive caregiving environment for children [29–31].

This pathway is supported by evidence from settings comparable to Colombia. A cross-national study spanning seven countries, including Colombia, found that economic hardship was linked to children’s adjustment through parents’ psychological distress and parenting [32]. In Colombia specifically, poverty, civil conflict, and domestic violence have each been associated with greater parenting stress and a higher likelihood of harsh discipline, partly through parents’ mental health [33,34]. These parenting practices, in turn, predict children’s own social and emotional development [3]. Together, this evidence outlines a chain in which poverty and hardship constrain parents’ social and emotional skills, shape nurturing parenting practices, and ultimately influence children’s social and emotional development. At the same time, it is important to recognize that many parents facing chronic stressors demonstrate remarkable resilience and adaptive capacity. Rather than being passive recipients of adversity, they often develop creative strategies to protect and support their children, adjusting their parenting practices and emotional responses to align with the specific needs of their families and the demands of their environment [35,36].

Colombia, an upper middle-income country, offers a compelling context for studying social and emotional skills due to its history of enduring a protracted civil conflict that spanned more than half a century. This ongoing conflict has heavily affected the mental health and social and emotional development of the Colombian population [37]. In 2016, Colombia signed a peace agreement with the largest guerrilla group in the country, The Revolutionary Armed Forces of Colombia (FARC), but the armed conflict and violence in Colombia persist, fueled by drug trafficking and violations of human rights [38].

Recent evidence shows that ongoing violence in Colombia continues to profoundly impact parenting and child development. For instance, in the municipality of Tumaco, where over 70% of the population is registered as victims of the civil conflict, exposure to recent violent events, combined with violence experienced by household members over the previous decade, has been linked to increased parenting stress, diminished nurturing behaviors, and heightened mental health problems in children [34]. These findings resonate with global evidence demonstrating that war exposure can undermine nurturing parenting with consequences for young children’s development and wellbeing [39].

Within such a challenging environment, the Government of Colombia has launched several strategies to support parents, promote child development, and raise a generation of children who contribute to peacebuilding. Among others, the Government launched in 2021 the National Pedagogical Strategy to Preventing Physical Punishment [40] and the Guidelines to Preventing Violence Against Children [13]. In both documents, the Government calls for more evidence and programs aimed at promoting nurturing parenting. Therefore, exploring the social and emotional skills parents view as important to provide nurturing parenting is especially timely in the Colombian policy context.

### The present study

This study aims to explore the social and emotional skills that parents in Colombia perceive as important for providing nurturing parenting through a descriptive qualitative approach [41]. Our specific research questions are: (1) What social and emotional skills do parents living in Colombia perceive as important for providing nurturing parenting? and (2) What risk and protective factors do they identify as influential in the development of these social and emotional skills? To address these questions, we use data from a two-stage qualitative process, including a broader study on violence prevention in the country and a targeted study focused on the social and emotional skills relevant to parenting. We employed a thematic approach to code skills focusing on three categories, i.e., emotional, social, and adaptability skills, based on social and emotional frameworks [21,42].

## Materials and methods

We followed the Consolidated Criteria for Reporting Qualitative Research (COREQ), a set of guidelines designed to ensure transparency and consistency in reporting of qualitative research studies, to develop this manuscript [43] (see S1 Table). We collected data in two phases. *Phase 1* was part of a broader study that investigated the perspectives of parents living in Colombia, program facilitators, and policymakers with the aim of informing the development of parenting programs to prevent violence against children in the country [44]. The current project uses data from the caregivers only, collected between April and May of 2022. *Phase 2* was a targeted study focused on the social and emotional skills parents living in Colombia identify as relevant to child-rearing. Data collection for this phase was collected in May 2024. All interviewers were members of the research team with prior training and experience in qualitative research and had completed the Collaborative Institutional Training Initiative (CITI Program) certification in human-subjects research. Information on each interviewer’s credentials, occupation, gender, and experience is provided in S2 Table.

### Researcher positionality

The research team comprises Colombian and international scholars from diverse disciplinary, geographic, and socioeconomic backgrounds. Some members have personally experienced poverty, forced displacement, or direct exposure to violence, while others acknowledge their relative distance from these realities and the ways privilege can shape their interpretation of the data. Throughout the group, there is a shared commitment to equity, reflexivity, and conducting research that informs policies that are inclusive, context-sensitive, and human-centered. Collectively, team members bring between approximately 5 and 25 years of experience working directly with caregivers of young children, and two members are mothers, drawing on their own caregiving experience. We mitigated potential bias through reflexive discussion among members with differing backgrounds, collaborative coding by multiple coders, and anchoring analytic decisions in participants’ own words.

### Participants

All participants were receiving services and programs offered by the Instituto Colombiano de Bienestar Familiar (ICBF), the public agency responsible for child and family policy in Colombia. To meet criteria to be beneficiaries, families have to be classified in the System of Identification of Potential Beneficiaries (SISBEN IV) as: living in extreme poverty, moderate poverty or vulnerable to falling into poverty [45]. For Phase 1, we selected three localities in the city of Bogotá, Colombia (i.e., Ciudad Bolívar, Kennedy, and Bosa), based on two indicators of vulnerability: 1) The number of reported child abuse cases for the year 2021 published by the Secretaría de Salud (Bogotá’s Department of Health); 2) The official poverty rates for children between the ages of 0 and 5 (i.e., households with a monthly income of less than $229.672 Colombian Pesos (approximately $53.41 USD) as reported by the Secretaría de Planeación (Planning Secretariat of Bogotá). The study involved 38 caregivers of children under five years old. For Phase 2, we targeted two localities in the city of Bogotá, Colombia (i.e., Mártires and San Cristóbal), chosen due to their high reported incidence of child abuse cases in 2023, as documented by the Secretaría de Salud (Bogotá’s Department of Health). It involved 16 caregivers of children under five years.

Table 1 provides an overview of the participants’ demographics, all of whom were parents of one to two children under five years. The selection criteria for Phases 1 and 2 were agreed upon through our research-policy partnership with the ICBF and responded to shifts in the institution’s prioritization mechanisms. These shifts reflected institutional priorities, particularly the need to address increasing rates of intrahousehold violence in the selected localities, which directly influenced the participant selection for Phase 2.

**Table 1:** Demographics of participants.

	Phase 1 (*n* = 38)	Phase 2 (*n* = 16)
Age, *M* [range]	27.8 [18–45]	33.6 [23–40]
Gender, *n* female (%)	37 (97.4%)	13 (81.2%)
Education, *n* (%)		
Less than High School	10 (26%)	2 (12.5%)
High School	20 (52%)	7 (43.7%)
Graduate’s degree (University Education)	8 (21%)	5 (31%)
		Post-graduate’s degree		1 (6.2%)
Number of Children, M [range]	1.28 [1 –2]	1 [1]	

Note: *M* = mean, *n* = number of participants.

### Measure and procedures

For ***Phase 1***, the ICBF randomly selected two or three service units in each locality (UDS, for their acronym in Spanish) in which families and children receive services. We launched two calls for participants through the ICBF over three weeks, including an online registration form with information on the study, and asked for sociodemographic data, locality, and consent. A total of 473 people registered, and 38 were randomly selected. The 38 participants were divided into two focus groups with a total of 18 participants (average of 9 participants per group) and 20 interviews. Focus groups lasted between one and 1.5 hours and were held at the ICBF’s service units during scheduled weekly workshops, while interviews were conducted by phone and lasted 15–35 minutes. The same researcher conducted all interviews and focus groups. No incentives were offered for participation.

Both interviews and focus groups followed a semi-structured format based on predefined themes. The research instrument addressed caregiver’s perspectives on violence prevention in the country and child-rearing practices and included questions such as: *How do you manage challenging behavior in your child? What do you say to your child when they misbehave?* And *what do you say to encourage positive behavior?*. (S3 Table presents a topic guide with sample questions for the instrument).

Given the sensitivity of some of the topics, we opted to conduct interviews and focus groups in parallel to triangulate data. This approach assumes that each method captures different aspects of the phenomenon, allowing for the exploration of both commonalities and contrasts in experiences, a deeper understanding of individual perspectives, and ultimately a more comprehensive and valid data collection process [46,47]. Families participating in interviews and focus groups shared common characteristics, particularly exposure to risk factors and vulnerability, providing a reliable source of data. Furthermore the use of both approaches allowed for varied insights: Individual interviews provided an in-depth exploration of personal experiences, whereas focus groups facilitated the emergence of additional perspectives through interaction among participants and the creation of a context that can encourage sharing similar or differing views [47]. All interviews and focus groups were audio recorded with participants’ consent.

For ***Phase 2***, we randomly selected two service units from each locality in collaboration with the ICBF. The ICBF contacted 28 caregivers to inform them about the study and invite them to participate. Caregivers were then called to confirm their willingness to participate and to schedule the interviews. 16 interviews were conducted over the phone, each lasting approximately 30 minutes. All interviews were audio recorded with participants’ consent.

Based on the codes generated during the first phase and our conceptual framework, we developed an instrument for the second phase. This instrument was designed to gather more detailed information on key themes related to social and emotional skills that were mentioned in the first phase but not explored in depth. The interviews covered emotional skills (e.g., “*What emotions do you feel most as a parent or caregiver?*”); social skills (e.g., “*How do you make child-rearing decisions with other caregivers?*”); and adaptation skills (e.g., “*How do you manage your time to fulfill these responsibilities in addition to child-rearing?*”). The questions were developed to allow respondents to construct their own interpretations and meanings regarding emotional, social, and problem-solving experiences. (See S4 Table for further details). Both instruments were piloted prior to each phase with caregivers who were not part of the study sample but were drawn from the same ICBF programs and comparable localities and met the same eligibility criteria, ensuring that the pilot groups were similar to the final samples.

The research was approved by the Institutional Review Board at Harvard University, and oral consent was obtained from all participants prior to data collection. In addition, the study underwent a technical approval process by the ICBF, which was required to contact and invite participating families. We took several measures to protect participants’ privacy and confidentiality. Telephone interviews were conducted individually, allowing participants to respond from a private setting of their choosing, and focus groups were held without non-participants present. Audio recordings and transcripts were stored on password-protected, access-restricted devices accessible only to the research team, and all transcripts were anonymized, with each participant assigned a numerical identifier. Interviewers followed a protocol to respond to participant distress, including pausing or ending the interview and referring participants to support resources.

### Data analysis

All interviews and focus groups were transcribed verbatim in Spanish by a native Spanish speaker. To ensure accuracy, all transcripts underwent a double-check process in which they were compared against the original audio recordings and any discrepancies were corrected prior to coding. The coding was done in two stages through the software NVivo 1.6.1. The first stage was done with data from ***Phase 1*** and followed a primarily inductive approach, following the method proposed by Braun and Clarke [48]. This method allowed us to focus on social and emotional skills while allowing for the natural emergence of themes. The coding was conducted by four researchers following a collaborative coding process [49], involving iterative phases of coding, codebook drafting, and re-coding, allowing for the refinement of the codebook and achieving agreement among coders. Prior to coding, all four coders were trained by a coding team member with extensive experience in qualitative research, who also reviewed the initial rounds of coding. To assess consistency, all coders independently coded a common subset of the data and, given the high level of agreement observed, the remaining transcripts were then divided among them. Coding decisions and discrepancies were discussed in regular team meetings and used to define each code with concrete examples in the codebook. Although not every transcript was independently double-coded, the codebook was developed collaboratively and refined through team consensus. The specific contributions of each team member at each stage of the study, including data collection and coding are detailed in S2 Table.

In the second stage, the same four researchers coded all interviews from Phase 2 and re-coded all interviews from Phase 1 using a mixed deductive and inductive approach to account for a more in-depth exploration of parents’ social and emotional skills. The progression from an inductive to a deductive method was implemented to integrate theoretical models with the lived subjective experiences of participants [50]. The final results reflect the refined topics and themes identified from both groups of data. Throughout the analysis, we retained divergent and minority perspectives alongside more commonly expressed views, and these are reported in the Results. In Phase 2, we considered that data saturation was reached at the point at which researchers began to hear repeated accounts and informational redundancy was perceived [51]. We selected representative quotes and translated them from Spanish to English after coding (see S6 Table for original Spanish quotes and their corresponding English translations). Findings were not returned to participants for validation, which was not feasible because interviews were conducted by telephone with participants drawn from different service units and localities and contact was mediated by the ICBF on a single occasion. The findings were, however, presented to the ICBF to inform the Apapacho parenting program and the institution’s broader programming.

Additional information regarding the ethical, cultural, and scientific considerations specific to inclusivity in global research is included in the Supporting Information (S7 File).

## Results

**Fig 1:**
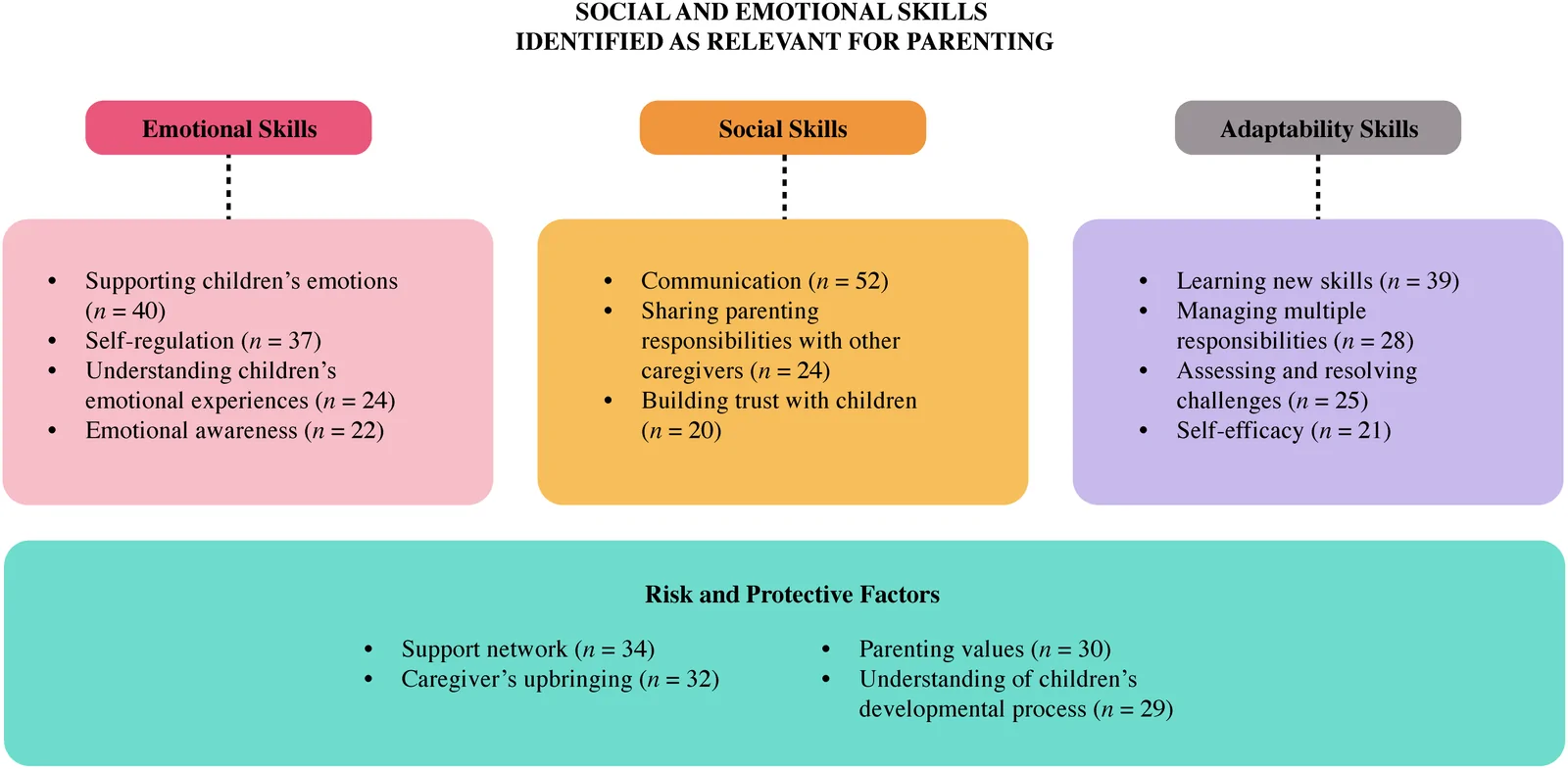
Coding tree: Social and emotional skills identified by parents as relevant to child-rearing. *n* represents the number of participants who reported the specific subtheme. *Note*. S5 Table presents a breakdown of topics, subthemes, and sample quotes. Original Spanish quotes and their translations are presented in S6 Table.

Fig 1 summarizes the main findings from this study. (see S5 Table for additional details).

Three central domains were defined by grouping the social and emotional skills parents identified as relevant to child-rearing (i.e., emotional skills, social skills and adaptability skills). These domains are a result of a deductive-inductive approach, in which theoretical frameworks informed the collection and initial data analysis, yet the final categories responded to participating parents’ perspectives and experiences. While there is a general correspondence between the proposed domains and different frameworks, these were defined not to respond to a specific theoretical model, but to be a reflection of this study’s participants’ lived experiences.

### Emotional skills

Four themes related to emotional skills emerged, organized from most to least mentioned by participants: a) Supporting children’s emotions b) self-regulation; c) understanding children’s emotional experiences; d) emotional awareness.

***Supporting Children’s emotions (n = 40).*** Caregivers described challenges and strategies for supporting their children’s emotions. Several participants mentioned the belief that attending to their child’s emotions could “*make them spoiled*”. One father said: “*If you scold him, and then go pamper him, he’ll do it again. So, I say, let him cry, so he won’t get used to it*” (Caregiver #4). In other cases, caregivers described not knowing how to accompany their child’s emotions, e.g., “*She has a really strong character, and it is difficult for me to educate her when she is so angry, because she is rebellious*” (Caregiver #13) or getting overwhelmed or angry, e.g., “*It makes me angry when he cries like that*” (Caregiver #1). In many cases, parents struggled to support their children’s emotions when disciplining them, or trying to teach them.

Moreover, caregivers described supporting their children’s emotions through various strategies. They soothed crying children by holding, hugging, or cuddling them, and talking to them lovingly: *“When they’re babies, pampering them, holding them, talking to them with love”* (Caregiver #17)*.* For younger children, breastfeeding was commonly used as a comfort method. Distracting children with games, or music, was also mentioned: *“When she is sad or crying, I hold her, I play music, songs I know she will like, or I give her rattles and she gets distracted”* (Caregiver #21)*.*

Caregivers supported their children through anger and frustration by talking to them and explaining things, e.g., *“I try to calm him down and I explain, depending on what happened (…) I tell him, I understand you’re angry, but you can do this or that”* (Caregiver #22)*.* They also described helping children if they were having difficulties with a task or giving them space to calm down*,* e.g., *“When he gets angry, he goes to a corner, and I try to give him space. So, he can go, and have some quiet, a moment of relaxation”* (Caregiver #25)*.* For these parents, accompanying their children’s emotions was a part of helping them solve problems or complete new tasks. For happiness and excitement, caregivers engaged by holding, hugging, and playing with children*,* e.g., *“Give them that same happiness, pampering them, hugging them”* (Caregiver #9)*.* Praising them was also mentioned, e.g., *“I celebrate him. If I see he’s making a puzzle and he’s happy because he finished it, then I clap, I congratulate him”* (Caregiver #27)*.*

***Self-Regulation (n = 37).*** Caregivers discussed challenges and strategies for regulating their own emotions. Participants described difficulties managing anger, which they related to “*being patient*”, and oftentimes led to recurring to physical punishment as a response to challenging behavior, e.g., “*I think I hit her out of anger*” (Caregiver #33). Thus, for some parents self-regulation appeared as a mediator for the style of discipline they used. They also described struggling with fear of their children getting hurt, e.g., “*That’s always been my fear, which I haven’t been able to control, being able to trust other people with he*r” (Caregiver #24). Difficulties with sadness and frustration over a sense of not having the necessary parenting skills were also mentioned, e.g., “*It makes me sad sometimes, I wonder what I should do to manage this, then I feel alone*” (Caregiver #26).

Furthermore, caregivers discussed different strategies they used to regulate anger, frustration and sadness, specifically, including breathing exercises, allowing themselves to feel the emotion, praying, distracting themselves, having time alone, seeking support, or reflecting on the causes of their emotions, or the negative consequences they might carry. For example, one participant said: “*Breathing deeply, because anger is mostly controlled with your breathing, and thinking about the consequences of the actions you might take*” (Caregiver #4). Another described: “*In that moment I prefer not to say anything, because I’m really explosive, so I prefer to calm down, give it some time, not talk to them, because I prefer to have my space and breathe, and then take action*” (Caregiver #11). The importance of “having patience” was commonly stated as important for not recurring to physical punishment when facing challenging behaviors.

***Understanding children’s emotional experience (n = 24).*** Caregivers noted the importance of recognizing and understanding the emotional experiences of children for their role as caregivers. One parent described it as: “*The dedication of knowing, of understanding what they want, what they need, what they want you to do with them*” (Caregiver #36). For example, caregivers mentioned knowing in what situations their child would feel angry or sad, and thus being prepared to react (“*When she is sick, she gets really spoiled, and she cries about everything, so I talk to her and then she calms down*,” Caregiver #3). In other cases, being able to “p*ut yourself in the child’s shoes*” (Caregiver #9) was described as a tool for self-regulation.

***Emotional awareness (n = 22).*** Most caregivers reported experiencing a range of emotions related to parenting, including happiness, anger, sadness, fear, frustration, and stress, leading the research team to identify emotional awareness as important. These emotions were named and elaborated upon in response to questions, and throughout their narratives of parenting. Caregivers described feeling angry when children do not follow their instructions, throw tantrums, or misbehave, mentioning behaviors such as hitting, throwing food, or refusing to eat. One participant said: “*It makes you angry when they don’t do what you say, that’s why sometimes you have to threaten them with the belt or the slipper, so they behave*” (Caregiver #5). Caregivers also expressed happiness when spending time with children, feeling their love, and seeing them grow and learn, e.g., “*He hugs me and gives me kisses, then that makes me happy*” (Caregiver #9).

Sadness was associated with family illness or death, separation, or violence, and how they impacted children, e.g., “*That makes me really sad, I tell you that really makes me feel bad, because it was not our fault his dad forgot about us*” (Caregiver #6). Economic hardships also caused frustration and sadness, as one caregiver mentioned: “*He asks for a lot of things and right now our economic situation is not one to give him everything. Yesterday he started asking for things, and I couldn’t get him what he asked for, so I felt sad*” (Caregiver #1). In other cases, participants described these feelings when they did not know how to manage certain behaviors or resorted to physical punishment, e.g., “*It hurt me more than it did him. (…) I felt frustrated because I thought I need to be able to know how to manage raising him without spanking*” (Caregiver #35).

Fear came up in the context of concerns over their children’s future, health, and safety, e.g., “*I think a lot about their future, how it’s going to be for them further on, that’s what I’m scared of*” (Caregiver #26). Finally, participants described feeling stress over having to manage multiple responsibilities, with one caregiver describing it as: “*Stress, as that sensation of not having enough time to do this, not knowing when you’re going to be able to do things*” (Caregiver #21).

Participants sometimes struggled to identify or name specific emotions in certain situations, describing feeling either “*good*” or “*bad*” or not finding words to describe their emotional experience. Furthermore, oftentimes the emotions identified by parents (i.e., anger, stress, fear) acted as mediators on how they managed caregiving, as for example in the case of anger or fear and the use of corporal punishment.

### Social skills

We identified the following key themes related to social skills, organized from most to least mentioned by participants: a) Communication; b) sharing parenting responsibilities with other caregivers; c) building trust with children.

***Communication (n = 52).*** Participants mentioned talking to their children as a response to behaviors considered either negative or dangerous by explaining things to them, as well as setting rules and consequences. One parent described: “*I have rules, and norms in the house, so she knows how far she can go. I talk to her*” (Caregiver #37). Another said: “*Say this week, her teacher came and said that he was misbehaving, so I talked to him and told him that he must respect his teacher and his mom*” (Caregiver #4). For dangerous behaviors, caregivers talked about describing the possible risks, because “*since they’re children, they don’t understand the consequences”* (Caregiver #4).

Caregivers also described giving children instructions, e.g., “*She plays and then we both pick up, I tell her, let’s pick them up*” (Caregiver #38) and talking them through all the activities they did, to help them understand and learn, e.g., “*Last night I told her we had to go to the doctor. So, I explained what the doctor was going to do to her. That’s what I always do*” (Caregiver #14). Participants also discussed teaching their children what’s good and bad: “*How do I want to teach her? Well, talking to her more every day, explaining to her how things are, what is good and what is bad. What is the right path, and what is the wrong path*” (Caregiver #8).

Finally, caregivers mentioned praising their children when they did something right or learnt a new skill. For example, one mother mentioned: “*We celebrate him a lot, because they say he is taking too long to speak. So right now, we are doing colors. He sees something blue, and he starts saying bl-, bl-, that means blue. So right now, we are praising him for that, so he feels safer to start saying things*” (Caregiver #37).

***Sharing parenting responsibilities with other caregivers (n = 24).*** Participants emphasized the importance of sharing responsibilities with other caregivers in child-rearing, emphasizing making decisions with other caregivers based on shared values and goals (“*We first see what’s right and what’s wrong, what we want to change or what we want to achieve for our daughters’ development and then we make decisions*” (Caregiver #8). Being able to manage conflict and not disagreeing with each other in front of children was also described, e.g., “*Say I didn’t like how he brought something up. But in front of the kids, we never fight, it’s after that I can go tell him I don’t agree*.” (Caregiver #21), as well as dividing tasks and coordinating schedules. They also mentioned the importance of asking for help or advice from their support network: “*There’s a lot of people that help you, for example, my brother, my brother-in-law, or even my wife. I’m not carrying all the weight myself, I call more people, and we manage it together*” (Caregiver #12).

Participants also described the challenges that came with a lack of coordination or support among multiple caregivers. Instances of one caregiver disagreeing with another were frequently mentioned: “*So when he’s here, he pampers her too much. So then when I go tell her she did something wrong, he tells me not to nag her, to be more patient. And that stresses me*” (Caregiver #36). Therefore, sharing responsibilities with other caregivers was a way of finding support for some parents, finding help managing stress and responsibilities, while for others it was a source of distress and conflict.

***Building trust with children (n = 20).*** Caregivers mentioned wanting their children to trust them, which they described as their child being comfortable talking to them, e.g., “*I want her to learn to trust me, to tell me anything, any time, under any circumstance*” (Caregiver #2). They believed trust was based on an open communication and offering support through problems, e.g., “*I always talk to him, and tell him that anything that happens, he can count on his dad, his mom and his brothers*” (Caregiver #1), as well as avoiding reacting with anger and yelling or hitting children when they come with problems, e.g., “*If he knows I’m going to get really angry because he took a toy to school, and I’m going to punish him, then he won’t tell me. So, I have to try and talk to them*” (Caregiver #9). Parents’ desire for building trust with their children acted as motivation for self-regulating and communicating, instead of reacting out of intense emotions or recurring to corporal punishment.

### Adaptability skills

Caregivers also identified adaptability skills as relevant to child rearing. Four major themes emerged from the data, organized from most to least mentioned by participants: a) learning new skills; b) managing multiple responsibilities; c) assessing and resolving challenges; and d) self-efficacy.

***Learning new skills (n = 39).*** Participants highlighted different knowledge and skills they wanted to learn in their role as caregivers. This included dealing with challenging situations (e.g., setting boundaries and consequences, managing tantrums) and learning appropriate games and activities for children. They mentioned seeking resources such as workshops, videos, books, and information through social media to learn, e.g., “*Reading books, and tutorials, I like seeking ways and resources, on how to help children”* (Caregiver #17), as well as seeking help from professionals (i.e., psychologists, nutritionists, doctors, etc.). As one caregiver described: “*I say it’s better to talk to someone who knows, not your friend (…) in such cases you need professional help*” (Caregiver #37).

***Managing multiple responsibilities (n = 28).*** Participants described the challenge of managing multiple responsibilities, including child-rearing, work, and home care, and the strategies they used to handle everything. Taking care of multiple children, or “*doing chores while with them*” (Caregiver #17) were highlighted as challenges. As for strategies, scheduling and time management were mentioned, as well as on help from family members or hiring help, with many participants leaving their children with relatives while they worked or attended to other tasks, e.g., “*I leave her with my mom, and since I don’t work all day, I work 3 or 4 hours, and then I come back and pick her up*” (Caregiver #8). Managing an overload of responsibilities was oftentimes related to stress, emotional dysregulation, and difficulty “having patience” with children.

***Assessing and resolving challenges (n = 25).*** Caregivers described coming up with creative solutions to challenging situations in parenting. Participants mentioned using games or songs to teach children skills or get them to do something, e.g., “*I’ve taught her to play “frozen”, so I say “frozen” and she stays still. And then I tell her “You’re frozen and I’m going to change your diaper*” (Caregiver #14). Using toys was also mentioned, as one mother talking about managing her child always taking her phone explained: “*Her dad got her one (a phone) that sings, so she plays with that*” (Caregiver #36). Coming up with consequences for challenging behaviors was also discussed by caregivers, including taking away access to mobile phones and/or the opportunity to go to the park.

***Self-efficacy (n = 21).*** For some caregivers, a sense of being capable of managing and overcoming difficult situations, even new ones, was highlighted “*Maybe I don’t have the knowledge, but when the challenge arises, you come up with something*” (Caregiver #8). Many described having certainty on their skills given prior experience raising their own older children or taking care of those of other family members. For other parents, the opposite was true as they described feeling overwhelmed over not having the knowledge or strategies to manage certain situations, e.g., “*Nobody gave me a script on how to be a dad. There is no manual*” (Caregiver #12), and thinking they’re “*bad parents.*”

### Protective/risk factors

A final theme that was apparent in the data referred to the factors that promote or hinder the development and use of parents’ social and emotional skills in the context of child-rearing.

***Support network (n = 34).*** Caregivers talked about relying on other people in their network to fulfill parenting tasks, as well as the difficulties of managing without help. The majority of participants described relying on partners, parents, siblings, older children, and neighbors to help them take care of child rearing responsibilities, including supervising children while the principal caregiver was working: “*My mom spends more time with the child than I do*” (Caregiver #6). On the contrary, other participants talked about the challenges of managing on their own after separating from their partners, e.g., “*We’ve been separated for a year and he has only had the boy two weekends*” (Caregiver #27) or moving countries, e.g., “*I’m Venezuelan (…) I felt alone, because I don’t have my mom or my sisters’ support*” (Caregiver #34).

The social support provided by caregivers’ networks plays a significant role in their ability to handle the stresses and responsibilities of parenting. This support not only contributes to caregivers’ emotional self-regulation but also provides them with the emotional resources needed to support their children effectively and engage in collaborative communication with other caregivers: “*Thanks to God my husband has always been here, he always gives me energy, he tells me: we can do this, we’re going to push through”* (Caregiver #25).

***Caregivers’ upbringing (n = 32).*** Participants discussed their own upbringings and how they had impacted their approach to child-rearing. Some caregivers discussed wanting to repeat their experience of nurturing and non-violent upbringing, e.g., “*My mom has always been loving and caring, so we’re like that, and I want my daughter to have that same upbringing*” (Caregiver #14), while others described seeking to break patterns of violence they lived in their own childhoods, e.g., “*I want to be different than my mom. With her, it was always about hitting us. I don’t want to be that way*” (Caregiver #36).

A final group of participants mentioned using physical punishment because they learnt it from their own parents, and believed it was a way to get their children to behave and respect them, e.g., “*You knew that if you did something wrong, then your dad would hit you, so you knew you had to do it well*” (Caregiver #4). In general, caregivers’ upbringing shaped their approaches to emotional awareness, regulation, and responsiveness toward their children. Participants who were raised in environments that promoted emotional expression and empathy reported better emotional awareness and self-regulation skills themselves, thus fostering similar skills in their children.

***Parenting values (n = 30).*** A final theme that impacted caregivers’ approach to child-rearing referred to the values they had and wanted to instill in their children. Multiple participants mentioned the idea that “*each one should educate their children how they want*” (Caregiver #6), which hindered their disposition to learn new skills. On the contrary, other caregivers demonstrated an openness to continuous learning and self-critique.

Furthermore, many caregivers highlighted the value of respect as something they wanted to teach their children. In some cases, respect was linked to authority and obedience, as one parent described when talking to spanking their child after a public tantrum: “*You have to make them respect you now, because later it becomes harder*” (Caregiver #5). In other instances, it was mentioned in relation to dialogue-based parenting practices aimed at fostering trust and emotionally supportive environments, e.g., “*Only educating children through dialogue, through respect regarding what’s right and what’s wrong, by explaining things to them*” (Caregiver #4). Moreover, participants mentioned other values they wanted to teach their children including: Politeness, patience, honesty, sociability, kindness, helpfulness, generosity, responsibility and gratefulness.

***Understanding of children’s developmental processes (n = 29).*** Caregivers described understanding that some challenges they faced with their child are a normal part of that process (e.g., babies making messes or having difficulty following instructions), which allowed them to exhibit greater patience and empathy, e.g., “*I try to stay very calm, because I understand that they’re just children”* (Caregiver #22). This awareness was also crucial for effectively managing their own emotions, supporting their children’s emotional development, building trust, and communicating effectively with children, e.g., “*so he can listen and start understanding, and he will keep growing on his time*” (Caregiver #34). Moreover, understanding the developmental process also enhances caregivers’ self-efficacy because they do not become frustrated by events they know to be normal in development, and have confidence in their ability to handle rather than eliminate them: “*I understand that he’s still small, and little by little he will learn*” (Caregiver #6).

## Discussion

This study explored the social and emotional skills that a group of parents living in Colombia identified as important to providing nurturing parenting. Sampled parents identified emotional skills that they consider important for parenting, including emotional awareness, self-regulation, understanding children’s emotional experiences, and supporting children’s emotions. These findings are consistent with the evidence indicating that emotional awareness, self-regulation [23,25,52], and understanding children’s emotional experiences or empathy predict parenting practices, including harsh punishment [27,52].

The study also revealed social skills that parents consider as important for parenting, including communication skills, sharing parenting responsibilities with other caregivers, and building trust with children as important. These results align with existing literature on how assertion, conflict management, and communication skills predict nurturing parenting and reduced parental stress overall [26,27]. These skills can be particularly relevant in Colombia, where families tend to be more extended and sharing parenting responsibilities is more common. Additionally, adaptability skills such as assessing and resolving challenges, learning new skills, managing multiple responsibilities, and self-efficacy were also identified as important. These skills have also been discussed in prior research and are thought to help parents manage responsibilities and parenting challenges, therefore motivating positive behavior [22–24,27].

The skills parents identified map onto established social and emotional learning frameworks while also extending them in context-specific ways. The emotional, social, and adaptability domains we used are aligned with the competencies described by CASEL [6], Jones et al. [19], and Soto et al. [21]. At the same time, parents foregrounded competencies that these frameworks tend to treat as secondary or implicit—most notably adaptability skills such as managing multiple responsibilities and self-efficacy—reflecting the demands of caregiving under economic and social adversity. Consistent with a bioecological perspective [14], this suggests that the salience of particular skills is shaped by context, and that frameworks developed in higher-income settings may underweight the adaptive competencies most relevant to caregivers in LMICs.

Our findings suggest that emotional skills, particularly emotion regulation, play a critical role in fostering parenting-related social and adaptability skills. Effective self-regulation appears to underpin the social and adaptability skills parents valued: by regulating their own emotions, caregivers described being better able to communicate calmly, sustain children’s trust through predictable responses, collaborate with other caregivers, and balance competing responsibilities. In this sense, emotion regulation functioned in parents’ accounts as a cross-cutting competency linking the three domains rather than as a separate skill. Overall, emotion regulation serves as a foundational element in cultivating a supportive family environment and enhancing children’s development and well-being. Supporting this, a meta-analysis of 53 studies by Zimmer-Gembeck et al. [25] found that parental emotion regulation significantly predicted nurturing behaviors, stress management, improved family functioning, and reduced aggression.

In addition to revealing the critical role of emotional, social, and adaptability skills for nurturing parenting, our results highlighted four protective factors that played an important role in enhancing these skills: Having a support network, understanding children’s developmental process, caregivers’ upbringing, and parenting values. Firstly, caregivers expressed that help from family members and neighbors was crucial to overcoming emotional hardships, assessing and resolving problems, learning new skills, and handling multiple responsibilities. Communication and coordination with multiple caregivers were central to child-rearing for most participants. These findings are consistent with an ecological perspective, which integrates the influence of factors spanning individual characteristics, family dynamics, and other factors in the broader context [14]. Prior research has also identified perceived social support as important for attenuating parenting stress, lessening the caregiving burden, and enhancing nurturing parenting [53]. Caregivers also mentioned that understanding developmental processes promoted empathy and emotional regulation in the face of daily challenges related to children’s normative behaviors.

Participants also identified their upbringing and parenting values as influential factors in shaping their emotional awareness, regulation, and responsiveness toward their children. Narratives involving contextual violence were a recurring theme, echoing previous research that links chronic exposure to violence with heightened parenting stress and diminished caregiving responsiveness [34]. These findings align with a recent meta-analysis indicating that caregivers who experienced adversity during childhood were often less emotionally available and more likely to adopt harsh or hostile disciplinary strategies, factors that negatively impact the emotional development of their children [54].

However, our findings also revealed contrasts with some prior evidence. A qualitative meta-synthesis by Herbell and Bloom [55] highlighted a “cycle-breaking” intention among parents who had experienced violence in childhood. These individuals consciously sought to cultivate nurturing relationships with their children as a way to counteract their own adverse experiences. Similar dynamics emerged in our data: While some participants acknowledged reproducing violent parenting practices, others emphasized a deliberate effort to provide their children with more positive and supportive childhood experiences than those they had endured.

Situating these findings within the regional and global evidence base highlights their relevance for parenting policy. Although parenting interventions in LMICs have shown benefits for children’s cognitive, language, and motor development, their effects on social and emotional outcomes have been weaker and less consistent [56], pointing to a need to ground social and emotional content more firmly in caregivers’ own perspectives. Yet relatively few parenting programs in these settings are developed locally or systematically informed by what parents already know, value, and do; most are adapted from models designed elsewhere [57]. Work with Latin American families similarly indicates that interventions are most acceptable when they explicitly engage local realities, including contextual stressors and culturally salient caregiving arrangements [58], and that frameworks emphasizing individual autonomy may not fit settings characterized by extended-family and collective caregiving [59], a pattern echoed in our participants’ emphasis on shared parenting. By documenting the skills Colombian caregivers themselves consider central to nurturing parenting, our study helps address this gap and provides a locally grounded basis for program design.

### Implications for policy and practice

Collectively, our findings and prior research indicate the need for incorporating more explicitly the development of social and emotional skills in policies and interventions aimed at supporting parents. While our study identified a broad range of important skills, the foundational role of emotional awareness and self-regulation suggests that these should be central components of effective parenting interventions.

Emotional skills not only enhance caregivers’ ability to manage their own emotions but also facilitate better understanding and responsiveness to children’s emotional needs, communication, and problem-solving skills [18]. These principles are reflected in the design of the Apapacho parenting program, which was informed by caregivers’ priorities and offers a concrete illustration of how locally grounded evidence can shape intervention content.

The emotional skills parents emphasized are addressed through sessions on adults’ emotion management and on recognizing children’s emotional experiences; their concern with responding to children’s distress without violence and with strengthening the caregiver–child relationship is reflected in content on supporting children’s emotions, managing tantrums nonviolently, and building a strong bond; and the value parents placed on communication is addressed in a dedicated session. Consistent with parents’ expressed need to feel capable, the program aims not only to build skills but also to strengthen caregivers’ self-efficacy. The strengths and needs families identified likewise shaped the program’s recruitment approach, helping present it as responsive to participants’ realities rather than externally imposed. This example illustrates a broader principle: anchoring parenting programs in caregivers’ own conceptualizations of relevant skills may improve their cultural relevance and acceptability.

Moreover, addressing factors that influence the development of skills in parenting programs, such as providing information on developmental processes and normal behavioral expectations for children at different stages, is crucial for promoting self-regulation, supporting children’s emotional needs, improving communication, and enhancing self-efficacy. Involving various family members in parenting programs, reinforcing the message of shared responsibility, and creating a supportive network can foster a collaborative approach to child rearing and enhance self-regulation. Finally, addressing caregivers’ adverse childhood experiences is critical to breaking intergenerational cycles of violence and supporting adult parents’ skill development.

### Limitations and future directions

This study has several strengths, such as the use of focus groups and interviews to triangulate the data, the use of social and emotional frameworks to guide data analysis, and the focus in an underrepresented context (i.e., Colombia). The study also has limitations. First, the study was conducted exclusively with families from urban areas in Bogotá who accessed ICBF services and resided in localities characterized by high economic vulnerability and elevated rates of violence against children, representing a significantly vulnerable population. Therefore, findings may be limited in generalizability, and future studies should include families from other cultural contexts, regions, and rural areas. Most participants were parents, but other significant family and adult caregivers were omitted, so future research should focus on integrating perspectives and experiences of non-parent caregivers, including grandparents, other family members, and even members of the community who may care for children.

Furthermore, Phase 1 of the current study was part of a broader study designed with a different purpose; accordingly, although we considered that data saturation was reached in Phase 2, saturation was not assessed in Phase 1 in relation to the present research questions, which may limit the richness of the Phase 1 data. Finally, future research could employ mixed-methods approaches to better understand the perspectives of parents and other adult caregivers regarding the social and emotional skills they would like to develop to inform parenting support programs and other programmatic efforts aimed at promoting the nurturing care of children.

## Conclusion

This study explored the social and emotional skills that parents in Colombia perceive as important for nurturing parenting, as well as the risk and protective factors that influence their development. Parents identified emotional awareness, self-regulation, dialogue-based communication, self-efficacy, sharing parenting responsibilities, managing multiple responsibilities, and assessing and resolving challenges as key skills that support nurturing parenting. These findings have important implications for policy and practice, highlighting the need for parenting programs to explicitly promote parents’ social and emotional skills. Additionally, fostering parent support networks and addressing contextual risk factors are critical for encouraging nurturing parenting and supporting children’s healthy development.

## Supporting information

S1 Table COREQ guidelines. Consolidated Criteria for Reporting Qualitative Research checklist detailing the methodological orientation, participant selection, data collection settings, and analysis framework used in the study.
(DOCX)

S2 Table Interviewer’s and coder’s positionality statements. Detailed reflexivity, academic credentials, and background profiles for each member of the research team.
(DOCX)

S3 Table Phase 1 interview and focus group guide sample questions. Topic guide and sample questions addressing caregiver’s perspectives on violence prevention in the country and child-rearing practices.
(DOCX)

S4 Table Phase 2 individual interview guide sample questions. Topic guide and sample questions addressing caregiver’s emotional, social, and adaptability experiences.
(DOCX)

S5 Table Codebook structure. Final codebook of socioemotional domains with their respective sub-themes, accompanied by exemplifying quotes.
(DOCX)

S6 Table Original quotes and translations. Original Spanish quotes included in the text and their corresponding English translations.
(DOCX)

S7 File Inclusivity in global research checklist. Completed checklist documenting the study’s approach to local research leadership, community engagement, and contextual relevance in accordance with journal requirements for research conducted in low- and middle-income countries.
(DOCX)
