# Supplementary material for: Social and emotional skills for nurturing parenting: A qualitative examination
Source: PLoS One. 2026 Aug 20;21(8):e0355716. doi: 10.1371/journal.pone.0355716 (PMC13492804; doi:10.1371/journal.pone.0355716)
Supplement: Supplementary file 1 [file pone.0355716.s001.docx]

**S1 Table:** COREQ Guidelines

| **Item** | **Guide Questions /Description** | **Page Number** |
| --- | --- | --- |
| ***Domain 1: Research team and reflexivity*** | | |
| 1. Interviewer/facilitator | Which author conducted the interviews? | *Table S3* |
| 2. Credentials | What were the researcher’s credentials? E.g. PhD, MD | *Table S3* |
| 3. Occupation | What was their occupation at the time of the study? | *Table S3* |
| 4. Gender | Was the researcher male or female? | *Table S3* |
| 5. Experience and training | What experience or training did the researcher have? | *Table S3* |
| 6. Relationship established | Was a relationship established prior to study commencement? | p. 10 *(research policy partnership with the ICBF)* |
| 7. Participant knowledge of the interviewer | What did the participants know about the researcher? e.g. personal goals, reasons for doing the research | p. 11 |
| 8. Interviewer characteristics | What characteristics were reported about the interviewer/facilitator? e.g. Bias, assumptions, reasons and interests in the research topic | *Table S3* |
| ***Domain 2: Study Design*** | | |
| 9. Methodological orientation and Theory | What methodological orientation was stated to underpin the study? e.g. grounded theory, discourse analysis, ethnography, phenomenology, content analysis | p. 9 |
| 10. Participant selection | Sampling How were participants selected? e.g. purposive, convenience, consecutive, snowball | p. 10 |
| 11. Method of approach | How were participants approached? E.g. face-to-face, telephone, mail, email | p. 11-12 |
| 12. Sample size | How many participants were in the study? | p. 10 |
| 13. Non-participation | How many people refused to participate or dropped out? Reasons? | Not recorded due to research-policy partnership. |
| 14. Setting of data collection | Where was the data collected? E.g. home, clinic, workplace | p. 11-12 |
| 15. Presence of non-participants | Was anyone else present besides the participants and researchers? | No. |
| 16. Description of sample | What are the important characteristics of the sample? E.g. demographic data, date | p. 10 |
| 17. Interview guide | Were questions, prompts, guides provided by the authors? Was it pilot tested? | p. 11-12 |
| 18. Repeat interviews | Were repeat interviews carried out? If yes, how many? | No. |
| 19. Audio/visual recording | Did the research use audio or visual recording to collect the data? | p. 11-12 |
| 20. Field notes | Were field notes made during and/or after the interview or focus group? | p. 12 |
| 21. Duration | What was the duration of the interviews or focus group? | p. 11-12 |
| 22. Data saturation | Was data saturation discussed? | Yes, reached in Phase 2. |
| 23. Transcripts returned | Were transcripts returned to participants for comment and/or correction? | No, due to research-policy partnership. |
| ***Domain 3: Analysis and Findings*** | | |
| 24. Number of data coders | How many data coders coded the data? | Table S3. |
| 25. Description of the coding tree | Did authors provide a description of the coding tree? | p. 13 |
| 26. Derivation of themes | Were themes identified in advance or derived from the data? | P. 12 - 13 |
| 27. Software | What software, if applicable, was used to manage the data? | p. 12 |
| 28. Participant checking | Did participants provide feedback on the findings? | No, due to research-policy partnership. |
| 29. Quotations presented | Were participant quotations presented to illustrate the themes / findings? Was each quotation identified? E.g. participant number | p. 14-23 |
| 30. Data and findings consistent | Was there consistency between the data presented and the findings? | p. 14-23 |
| 31. Clarity of major themes | Were major themes clearly presented in the findings? | p. 14-23 |
| 32. Clarity of minor themes | Is there a description of diverse cases or discussion of minor themes? | p. 14-23 |
