# Supplementary material for: Social and emotional skills for nurturing parenting: A qualitative examination
Source: PLoS One. 2026 Aug 20;21(8):e0355716. doi: 10.1371/journal.pone.0355716 (PMC13492804; doi:10.1371/journal.pone.0355716)
Supplement: Supplementary file 2 [file pone.0355716.s002.docx]

**S2 Table:** Interviewer’s and Coder’s positionality statements

| **Researchers** | **Initials** | **Participation** | **Positionality Statement** |
| --- | --- | --- | --- |
| Researcher 1 | LB | Conducted all interviews and focus groups for ***phase 1***.  Conducted part of the interviews for ***phase 2***.  Participated in both phases of coding.  Participated in conceptualization and writing. | I am a Colombian cisgender woman with a bachelor’s degree in psychology and master’s degrees in sociology and political science, and with professional experience in political communications and content creation, I approach research with the goal of understanding how personal experiences and societal structures interact to shape individual possibilities and social realities. Coming from an upper-class urban background, with no personal experience of violence or displacement, yet having worked for several years with families in vulnerable contexts, participated in political campaigns across the country, and supported the passage of the socioemotional education law in Congress, I am deeply aware of the structural divides (particularly those related to class and exposure to violence) that influence caregiving, parenting practices, and the development of socioemotional skills. Through my previous and current work with caregivers, I approach research with the curiosity and humility of knowing that the each person knows their own story their best. I try to reduce researcher´s bias through deep listening and radical curiosity. |
| Researcher 2 | JB | Conducted part of the interviews for ***phase 2***.  Participated in both phases of coding.  Participated in conceptualization and writing. | I am a Colombian cisgender woman with a degree in Government and Public Affairs. I come from a family that overcame economic hardship through the opportunities afforded by public higher education. This background has shaped my appreciation for the role of education in breaking cycles of inequality and fostering socioemotional well-being. My work as a school teacher in Malambo, a vulnerable municipality in the Colombian Caribbean, brought me close to multiple forms of violence—state neglect, drug-related killings, and domestic abuse—which deeply influenced my understanding of how adversity undermines socioemotional life and reinforces structural injustice. These experiences have shaped not only my commitment to addressing socioemotional inequities, but also to challenging the systems of oppression that sustain them. Over the past five years, I have worked directly with caregivers as a public servant, a school teacher, and now through Fundación Apapacho. |
| Researcher 3 | JC | Participated in conceptualizaton, data analysis, review and editing | I am a Colombian male with a bachelor's degree in Economics, master's degrees in Economics and in Psychology and Human Development, and a Ph.D. in Education. I am currently an Assistant Professor at a U.S.-based institution, focusing on the prevention of violence against children and the promotion of early childhood development. Coming from a low-income urban background and a family with a history of displacement, I am aware of structural inequities. My personal experiences shape my commitment to advancing equity through policy, practice, and research. I have over eight years' experience leading Fundación Apapacho, a Colombian NGO promoting early childhood development, and more than five years working with caregivers of children aged 0–6 in government-led violence-prevention initiatives. |
| Researcher 4 | CRG | Participated in conceptualizaton, data analysis, review and editing | I am a feminist cisgender scholar committed to examining how social norms and power structures shape caregiving practices and family well-being. Born and raised in Colombia, I have direct experience with the cultural and socioeconomic contexts that influence parenting beliefs and practices in a middle-income country setting. As co-founder and co-director of Fundación Apapacho, I have worked closely with families and caregivers, centering their perspectives to promote children’s and caregivers’ social and emotional well-being. This applied experience informs my understanding of how caregivers conceptualize and develop skills such as emotional regulation, communication, and cooperation within everyday family life. During my doctoral training in developmental psychology in the United States, I engaged with qualitative and feminist research approaches that emphasize participants’ lived experiences and the co-construction of knowledge. These perspectives shape my approach to this study, guiding me to foreground caregivers’ own voices while critically reflecting on how knowledge about parenting and development is produced, particularly in low- and middle-income country contexts. |
| Researcher 5 | MAG | Conducted part of the interviews for ***phase 2***.  Participated in both phases of coding.  Participated in reviewing and editing. | As a Colombian cisgender woman, physician, and researcher working in child development, mental health, and family well-being, my professional experiences have deeply shaped my perspective on caregiving. Through my work with Fundación Apapacho I collaborated with caregivers facing displacement, migration, poverty, and other crisis-related stressors, including families crossing the Darién Gap and internally displaced families in Colombia. I have also worked with refugee and migrant children and families through programs at Yale and Boston College, as well as with communities affected by conflict and adversity in Colombia and other low-resource settings. These experiences have allowed me to observe both the challenges caregivers face and the extraordinary strengths they draw upon to support their children under difficult circumstances. |
| Researcher 6 | MG | Conducted part of the interviews for ***phase 2***.  Participated in both phases of coding.  Participated in reviewing and editing. | As a Colombian cisgender woman with a background in psychology and a Master's degree in Childhood, Family, and Development in Contexts, my personal, academic, and professional trajectory has profoundly shaped my interest in social-emotional skills and their role in parenting and caregiving processes. Since adolescence, inspired and supported by my mother, I participated as a volunteer working with children and their families. These early experiences allowed me to recognize how the ways caregivers identify, express, and regulate their emotions directly influence the relationships they build with their children and, at the same time, shape the emotional strategies that children will carry throughout their lives. Later, my academic training and professional experience reinforced this understanding and provided me with tools to promote social-emotional skills across different intervention settings. Additionally, my personal experience as the mother of a young child has confronted me directly with the everyday challenges of parenting. This experience has helped me recognize that identifying one’s own emotions is only a first step and that tools, resources, and ongoing practice are needed to navigate the emotional demands of caring for and supporting a child. At the same time, I have witnessed the value of emotional validation, respectful guidance, and the teaching of simple strategies, such as breathing techniques, in supporting my child’s emotional development. These experiences have strengthened my conviction that social-emotional skills are protective factors against multiple social challenges and essential tools for violence prevention. This perspective informs my approach to research and interventions, as well as my interest in understanding and strengthening the emotional capacities of those involved in caregiving and parenting processes. |
| Researcher 7 | HHB | Participated in conceptualizaton, data analysis, review and editing | I am a white, cisgender middle-class, Welsh, female professor. I have a PhD related to early childhood development and have worked extensively with families, teachers, and health care staff in the majority world. This includes working in contexts in which children are exposed to high levels of violence both at home and at school and in the community. I am aware that my positionality as a professor, my professional experience, and my background can influence how I interpret, and present research findings and I strive to be self-reflective and transparent in my research. I have 25 years' experience in early childhood research programmes across the Global South (Indonesia, Bangladesh, Jamaica, and Colombia), including over five years working with caregivers in violence-prevention interventions in Jamaica. I am also a mother of two now-adult, mixed-race (Welsh-Jamaican) children raised across Jamaica and Wales, which gives me personal experience of cross-cultural caregiving |
