# Supplementary material for: Social and emotional skills for nurturing parenting: A qualitative examination
Source: PLoS One. 2026 Aug 20;21(8):e0355716. doi: 10.1371/journal.pone.0355716 (PMC13492804; doi:10.1371/journal.pone.0355716)
Supplement: Supplementary file 3 [file pone.0355716.s003.docx]

**S3 Table**: Phase 1 interview and focus group guide sample questions

| Topic | Questions |
| --- | --- |
| Introduction questions  Attitudes towards physical punishment  Perceptions of children’s behaviors  Child behavior challenges  Strategies to manage child behavior challenges  Strategies to reinforce positive child behaviors  Sources of information for parenting  Relationships and interactions | Think about your role as a parent or caregiver and what things you enjoy the most and which are more complicated. Let us start by talking about the things you like. Now tell me about some more difficult things.  When you think about disciplining children, what comes to mind?  When you think about physical punishment, what comes to mind?  What are some of the reasons why parents and/or caregivers in your community use physical punishment, like spanking, pinching, or hitting?  What do you think parents hope to achieve by using physical punishment?  What do you think about spanking, pinching, or hitting children as a method for them to learn how to behave?  Should mothers/fathers/caregivers be the only ones to decide whether to use spanking, pinching, or hitting, or other forms of physical punishment to discipline children? Why or why not?  Think about the times when your son or daughter behaves well. Tell me what your child does and says in those cases.  Can you give me some examples of behaviors of your son or daughter that you find irritating?  Can you give me some examples of children's behaviors that you find completely unacceptable?  What are the main problems you have with your son or daughter?  What do you do when your son or daughter has these behaviors?  Have you ever responded to these behaviors by yelling, spanking, pinching, or hitting your child? In what case?  How do you encourage your child to behave well?  What are the main sources (for example, television, radio, neighbors, family, teachers, ICBF) from which you get information about parenting and disciplining children?  Think about your role as a parent or caregiver, what things you enjoy most and which are more difficult. Let us start by talking about the things you like. Now tell me about some things that are more difficult.  What does your child play with and what toys does he or she play with?  What kind of activities or games do you usually do with your child?  What does a normal day look like for you and your child? |
