# Supplementary material for: Social and emotional skills for nurturing parenting: A qualitative examination
Source: PLoS One. 2026 Aug 20;21(8):e0355716. doi: 10.1371/journal.pone.0355716 (PMC13492804; doi:10.1371/journal.pone.0355716)
Supplement: Supplementary file 4 [file pone.0355716.s004.docx]

**S4 Table**: Phase 2 individual interview guide sample questions

| Topic | Questions |
| --- | --- |
| Emotional skills  Social Skills  Adaptation and problem-solving skills | In the context of parenting, what are the emotions you feel the most? In what situations do you feel each emotion?  Think about the last time you felt X. What happened? How did you feel? How did you handle it? Do you always react like that? Do you think that's the best way to react? If not, how would you like to react to that feeling?  In the context of parenting, what emotions do you think your child feels the most? At what moments have you identified that they feel this way? How do you react when your child feels that way? Do you always react like that? Do you think it's the best way to react?  Other caregivers have mentioned the importance of building trust with children. Is this important to you? How is this trust built?  Do you share parenting tasks with other caregivers? If so, who are they?  How do you coordinate parenting responsibilities with these individuals?  How would you describe the communication with these individuals?  What is the process of making decisions about parenting with other caregivers like?  How do you resolve problems with other caregivers regarding parenting?  Do you have additional responsibilities besides parenting? If so, what are they?  How do you manage your time with these responsibilities?  Think about times when you are overwhelmed with responsibilities. How do you handle it? Do you ask for help from others? Do you always handle it this way? Do you think this is the best way to do it?  Do you feel you have enough knowledge and skills to handle the various challenges involved in parenting?  What skills or knowledge would you like to strengthen or acquire? |
