# Supplementary material for: Social and emotional skills for nurturing parenting: A qualitative examination
Source: PLoS One. 2026 Aug 20;21(8):e0355716. doi: 10.1371/journal.pone.0355716 (PMC13492804; doi:10.1371/journal.pone.0355716)
Supplement: Supplementary file 5 [file pone.0355716.s005.docx]

**S5 Table:** Codebook Structure

| Topics | Sub-themes | Sample Quotes |
| --- | --- | --- |
| Emotional Skills  Social Skills  Adaptation and Problem Solving Skills  Protective/Risk Factors | Emotional awareness  Self-regulation  Understanding children's emotional experiences.  Supporting children´s emotions  Communication  Shared parenting responsibilities with other caregivers  Building trust with children.  Assessing and resolving challenges  Learning new skills  Managing multiple responsibilities  Self-efficacy  Understanding of children´s developmental process  Support network  Caregivers’ upbringing  Parenting values | “*It hurts me when I hit him, because it really hurts me more than it does him. It makes me feel guilty. Like, I hit him, now what am I supposed to do?.*” (Caregiver #6).  “*When I´m really angry, I start crying, or else I get red*.” (Caregiver #1).  “*I think sometimes you have to calm down, breathe, and realize what is really going on.”* (Caregiver #8).  “*You must breathe, calm down, when you feel like you can´t handle it any longer. Because it’s not their fault that you're stressed, that you´re tired*.” (Caregiver #40).  “*He gets altered; he cries. And it’s not bad to cry, because when you get angry or sad, you cry, and it´s not bad*”. (Caregiver #27).  “*If she cries it´s because she feels that I´m leaving. It´s that distance because I must leave her while I go work*”. (Caregiver #21).  “*When I had the girl, she cried a lot, and I didn´t know what to do, so I cried, because I didn´t know*” (Caregiver #19).  “*I explain why he shouldn´t do those things, and try to calm him down, help him channel that emotion*.” (Caregiver #35).  “*You have to teach your children by talking to them and putting things in practice*.” (Caregiver #8).  “*When he misbehaves, I scold him, as in I ask him: what´s happening to you? What do you want? Things like that, I talk to him.”* (Caregiver #34).  “*If I notice I´m overloaded and I have something I can´t accomplish, I communicate that to my wife and see what agreement we reach*” (Caregiver #12).  “*When her mom is not home, I take care of the girl, and when we´re both home, when her mom has time, I work and she takes care of the girl*.” (Caregiver #2).  “*I want him to see me as a person that he can trust in, who won´t judge him, or whatever he´s going through, any problem or situation. So, I have to build that trust, through love, respect and quality time*.” (Caregiver #35).  “*I think that to build trust, the first thing you must do is listen. Listen to him, try to understand him, put yourself in his shoes*.” (Caregiver #25).  “You incentivize them *yhrough children´s music.Let´s go eat, let´s go play. It´s a way to do it*.” (Caregiver #29).  “*We do races to see who wins, when we organize. Or we tell him, if you organize, you can sleep in our bed.”* (Caregiver #16).  “*I watch parenting videos on how to manage situations. Because sometimes because you´re angry, you make the mistake of hitting them. So, it´s about learning how to manage the situation*.” (Caregiver #32).  “*I learnt that at a foundation where they teach all those things, they give us workshops, on what you should do, and how. Sometimes you also read things, and that also helps you be a better parent*.” (Caregiver #29).  “*At work, right now I´m the boss and it´s a little hard. And at home, with my husband, because he´s like another child*.” (Caregiver #35).  “*It is difficult, because you must be a dad and a mom at the same time. You must watch after him, but also take care of work*.” (Caregiver #23).  “*I really like educating my children. My neighbors talk well about my children, they say: your children are very well educated, very respectful. I was raised that way, so I like it when they say that about my kids. I feel proud, it´s a blessing to me*.” (Caregiver #34).  “*I felt happy, because I see my son´s progress, and I know that he's a very smart boy, and that he is learning very quickly. And that satisfies me, because I know I´m doing things right*.” (Caregiver #22).  “*He is still very little, he doesn't even know how to hold a spoon, or he takes it to play with it. So you understand he will spill, because he doesn´t even know how to hold a plate*.” (Caregiver #33).  “*She is still a child, and she will cry because she needs something, her bottle or anything. I try to be conscious of that, because she is a child, and she will ask for her needs, and as a mother, I must attend to them*.” (Caregiver #30).  “*I seek my brother, to tell him things, to vent. You know, when you talk about things, you unload*.” (Caregiver #35).  “*At work, if I need to get out earlier for any reason, I talk to my boss. And at home, if I need help with anything, I ask my mom or my husband*.” (Caregiver #22).  “*The way my parents raised me wasn´t the best, that is why I try not to do the same with my daughter. I want to change those things I saw were bad*.” (Caregiver #30).  “*Sometimes you want to hit them, but I try to relax. I tell myself, I won´t hit him, because that´s not what my parents taught me*.” (Caregiver #19).  “*An educated girl is respectful. She greets people: good morning, good afternoon, good night. If you tell her something, she won´t respond harshly*.” (Caregiver #11).  “*For them to be well behaved, not to be annoying or rude. Showing them what´s good or teaching them by example*.” (Caregiver #5). |
