# Supplementary material for: Social and emotional skills for nurturing parenting: A qualitative examination
Source: PLoS One. 2026 Aug 20;21(8):e0355716. doi: 10.1371/journal.pone.0355716 (PMC13492804; doi:10.1371/journal.pone.0355716)
Supplement: Supplementary file 6 [file pone.0355716.s006.docx]

**S6 Table:** Original Quotes and Translations

| Theme | Caregiver | Original Quote | Translated Quote |
| --- | --- | --- | --- |
| ***Emotional Skills***  Supporting Children's Emotions  Self-Regulation  Understanding children’s emotional experience  Emotional awareness  +  *Social Skills*  Communication  Sharing parenting responsibilities with other caregivers  Building trust with children  *Adaptability Skills*  Learning new skills  Managing multiple responsibilities  Assessing and resolving challenges  Self-efficacy  *Protective / Risk Factors*  Support network  Caregivers’ upbringing  Parenting values  Understanding of children’s developmental processes | Caregiver #4  Caregiver #13  Caregiver #1  Caregiver #17  Caregiver #21  Caregiver #22  Caregiver #25  Caregiver #9  Caregiver #27  Caregiver #33  Caregiver #24  Caregiver #26  Caregiver #4  Caregiver #11  Caregiver #36  Caregiver #3  Caregiver #9  Caregiver #5  Caregiver #9  Caregiver #6  Caregiver #1  Caregiver #35  Caregiver #26    Caregiver #21  Caregiver #37  Caregiver #4  Caregiver #4  Caregiver #38  Caregiver #14  Caregiver #8  Caregiver #37  Caregiver #8  Caregiver #21  Caregiver #12  Caregiver #36  Caregiver #2  Caregiver #1  Caregiver #9  Caregiver #17  Caregiver #37  Caregiver #17  Caregiver #8  Caregiver #14  Caregiver #36  Caregiver #8  Caregiver #12  Caregiver #6  Caregiver #27  Caregiver #34  Caregiver #25  Caregiver #14  Caregiver #36  Caregiver #4  Caregiver #6  Caregiver #5  Caregiver #4  Caregiver #22  Caregiver #34  Caregiver #6 | “Es que, si uno lo regaña y va y lo consiente, va y lo vuelve a hacer, entonces yo le digo que llore un poquito pa’que no, pa¨que no vaya a cogerlo como de costumbre, pué, digamos”  “Es de un carácter muy fuerte y pues para mí, digamos, para mí educarla así sobre ese mal genio es un poquito complicado, si porque ella es rebelde, por lo que es tan consentida es rebelde.”  “Si, a mi me molesta que él llore así.”  “Cuando son bebés, mimarlos, alzarlos, hablarles con cariño.”  “Si, cuando ella está triste o llorando y eso, yo la alzo, le pongo música, le pongo las canciones que yo sé que a ella le gustan, le paso los sonajeros.”  “Trato de tranquilizarlo y le explico, digamos dependiendo de lo que pasó (...) pues le digo, también entiendo que estas enojado pero puedes hacer esto o esto, y ya.”  “Cuando él se enoja se va al rinconcito y se trato de darle su espacio, que él se quede allí y hacer silencio, que tenga su momento de relajación.”  “Darle la misma alegría, consentirlo, abrazarlo, mimarlo.”  “Le celebro lo que él… pues si yo veo que está armando un rompecabezas y esta feliz porque lo terminó, lo armó, entonces lo aplaudo, lo felicito, asi.”  “Yo creo que yo ya le pego es de rabia.”  “Yo digo que mi miedo es ese de dejarla, de soltarla a ella si, pes a otras personas, ese siempre ha sido mi miedo, mi tema, que no he sabido manejar, esa confianza a las personas no no la tengo.”  “Me da como también me da como tristeza a veces, me pregunto cómo qué hago yo para manejar esto… entonces me siento como sola.”  “Respirando profundo, porque la rabia se, se controla más que todo con la respiración y pensando qué consecuencias pueden traer los actos que uno haga.”  “En esos momentos ee pues ya en el momento en que realmente pasa, prefiero yo no decir nada, no decir nada, porque yo realmente soy muy explosiva, entonces prefiero calmarme, esperar un tiempo, no hablar ni con ellos porque prefiero que me den mi espacio y respirar, y ahí si tomar acciones.”  “La dedicación de saber uno, de entenderles lo que ellos quieren, lo que necesitan, lo que, lo que ellos quisieran que uno haga con ellos.”  “Cuando ella está enfermita por ejemplo se pone muy consentida, y ella por todo quiere llorar entonces, yo como que le hablo y eso y ella como que se calma.”  “Tratar un rato de ponerse en el lugar de ellos.”  “Da rabia a veces cuando no hacen caso, por eso es que hay que amenazarlos a veces con la correa o con la chancla para que ellos hay que si hagan caso.”  “Cuando él llega, me abraza, él llega y me besa sin yo pedírselo, eso me causa mucha alegría.”  “A mi me da mucha tristeza eso, mira, yo te cuento eso y a mí eso de verdad me pone mal porque está bien, nosotros no tuvimos culpa porque el papá se haya olvidado de nosotros.”  “Él pide muchas cosas y ahorita la situación económica no está para complacerlo en todo, ayer empezó a pedir cosas y cosas y cosas, y no podía darle lo que me pedía, entonces ahí estaba como triste, me hace sentir triste.”  “Me dolió más a mí que a él (...) Entonces ahí me sentí frustrada porque dije noooo yo tengo que que poder saber como hacer con la con la crianza de él sin necesidad de usar de pronto las palmadas o así…”  “Yo si pienso mucho en el futuro de ellos, cómo les va tocar un poco más adelante, a eso le tengo miedo.”  “El estrés, como esa sensación de que el tiempo no me va a alcanzar para hacer esto y cuando me puedo dedicar a hacer esas cosas.”  “Manejo reglas, manejo normas en la casa y ella sabe hasta qué punto, puede, puede uno llegar, yo le hablo.”  “En éstos días la maestra me dijo que él llegaba y se quería portal mal, que no quiere hacer caso y yo hablo con él y le digo que tiene que respetar a la maestra y a la mamá.”  “Como son niños no saben las consecuencias.”  “Ella juega y después nos ponemos las dos y recogemos, yo le digo, vamos a recogerlo.”  "Desde anoche le decía que teníamos que ir al médico, entonces le explicaba que le iban a hacer en el médico, entonces yo casi siempre le comento a ella todo."  “¿Cómo le quiero enseñar? Pues cada día hablándole más, diciéndole explicándole cómo son, que es bueno, qué es malo, qué camino es bueno, qué camino es malo.”  “Le celebramos mucho, por lo que dicen que él se está demorando mucho en hablar, entonces ahora estamos con los colores, él ve algo azul, todo lo que ve que es azul dice abu, abu, ese es azul y por eso, eso es lo que estamos aplaudiendo ahora, para que él se sienta como más seguro para poder decir algo.”  “Nosotros primero colocamos lo que está bien, lo que está mal, lo que queremos cambiar o lo que queremos implementar en el crecimiento y desarrollo de nuestras hijas y ahí ya tomamos una decisión.”  “Que no me gusto como llamó la atención o algo, entonces lo que si e que siempre frente a las niñas, somos la pareja de nunca pelear, pero ya después, no es que a mi no me pareció esto.”  “Hay muchas personas que lo ayudan a uno, ejemplo, mi hermano, mi cuñado, un primo, mi misma esposa, yo no cargo con la bola de nieve yo mismo, yo llamo más gente y entre todos la picamos y entre todos la deshacemos.”  Entonces los días que él está, la consiente demasiado, entonces ya eso sí, ya la voy a regañar, entonces él, que no la regañe, que mire tengale paciencia, entonces eso me estresa.”  “Que ella aprenda a confiar en mí, aprenda a contarle a uno todo en cualquier momento y en cualquier circunstancia.”  “Trato de hablar bastante con él y decirle que me cuente cualquier cosa y que cuente con su papá y su mamá, sus hermanos.”  “Si él sabe que yo me voy a poner super brava porque llevó un juguete y lo voy a regañar, pues él no me va a decir, pero entonces debo tratar es como de hablarles.”  “Leyendo libros, tutoriales, me gusta más que todo buscar formas, recursos, en tanto cómo, como ayudar tanto a los niños.”  “Con alguien que realmente sepa, que no que es que la amiga (...) no, yo digo, que uno necesita ayuda profesional en esos casos.”  “La más retadora es hacer oficio con él.”  “La llevo donde mi mami, igual yo no trabajo todo el día cuando estoy trabajando, yo trabajo tres, cuatro horas, entonces de ahí vengo la recojo.”  “Digamos yo la he enseñado a jugar a los congelados, entonces le digo “congelada”, entonces ella se queda ahí,  entonces yo le digo “tú estás congelada y te voy a cambiar el pañal”.”  “El papá le consiguió uno que canta entonces ella mantiene con su teléfono.”  “Igual de pronto no tenga el conocimiento, pero en el momento de la dificultad uno le sale conocimiento de donde no lo tiene para ponerlo en práctica.”  “Porque el libreto de padre no venía, ¿no? no hay como los manuales de instrucción.”  “Pasa más tiempo mi mamá que yo con el niño.”  “Llevamos un año separados y se ha quedado con el niño dos fines de semana no más.”  “Uno es venezolano (...) pero yo me sentía sola, porque no tengo a mi mamá, no tengo el apoyo de mis hermanas.”  “Gracias a dios mi esposo siempre está aquí, entonces siempre me da energía, comienza no amor nosotros podemos vamos a hacer esto, vamos a salir de esto.”  “Mi mamá ha sido cariñosa, es muy atenta, es muy amable, digamos nosotros somos así y por ende pues yo quiero que mi hija que esta chiquitina, pues también crezca como, quiero que no se pierda como ese, esa tradición.”  “Yo quiero actuar como diferente a mi mamá, mi mamá a toda hora eran golpes a uno, a toda hora por todo lo golpeaba a uno, yo nunca he querido ser así.”  “Antes, uno sabía que si hacía algo mal, decía, mi papá me va a pegar, entonces tengo que hacerlo bien.”  “Eso ya es como cada quien lo maneje.”  “Desde ya hay que hacerse respetar porque cuando ya estén grandes ya es difícil.”  “Solo educar a los hijos a través del diálogo de la, a través del respeto de saber lo que está bien, lo que está mal, explicándole a sus hijos.”  “He tratado de estar siempre muy tranquila con las cosas de los niños porque pues también entiendo que son niños.”  “Pa que vaya escuchando y vaya entendiendo lo que uno le está, para que en su su tiempo que él vaya creciendo.”  “Yo entiendo, él está pequeño pero que poquito a poco vaya aprendiendo.” | “If you scold him, and then go pamper him, he’ll do it again. So, I say, let him cry, so he won’t get used to it”  “She has a really strong character, and it is difficult for me to educate her when she is so angry, because she is rebellious.”  “It makes me angry when he cries like that.”  “When they’re babies, pampering them, holding them, talking to them with love.”  “When she is sad or crying, I hold her, I play music, songs I know she will like, or I give her rattles and she gets distracted.”  “I try to calm him down and I explain, depending on what happened (…) I tell him, I understand you’re angry, but you can do this or that.”  “When he gets angry, he goes to a corner, and I try to give him space. So, he can go, and have some quiet, a moment of relaxation.”  “Give them that same happiness, pampering them, hugging them.”  “I celebrate him. If I see he’s making a puzzle and he’s happy because he finished it, then I clap, I congratulate him.”  “I think I hit her out of anger.”  “That’s always been my fear, which I haven’t been able to control, being able to trust other people with her.”  “It makes me sad sometimes, I wonder what I should do to manage this, then I feel alone.”  “Breathing deeply, because anger is mostly controlled with your breathing, and thinking about the consequences of the actions you might take.”  “In that moment I prefer not to say anything, because I’m really explosive, so I prefer to calm down, give it some time, not talk to them, because I prefer to have my space and breathe, and then take action.”  “The dedication of knowing, of understanding what they want, what they need, what they want you to do with them.”  “When she is sick, she gets really spoiled, and she cries about everything, so I talk to her and then she calms down.”  “Put yourself in the child’s shoes.”  “It makes you angry when they don’t do what you say, that’s why sometimes you have to threaten them with the belt or the slipper, so they behave.”  “He hugs me and gives me kisses, then that makes me happy.”  “That makes me really sad, I tell you that really makes me feel bad, because it was not our fault his dad forgot about us.”  “He asks for a lot of things and right now our economic situation is not one to give him everything. Yesterday he started asking for things, and I couldn’t get him what he asked for, so I felt sad.”  “It hurt me more than it did him. (…) I felt frustrated because I thought I need to be able to know how to manage raising him without spanking.”  “I think a lot about their future, how it’s going to be for them further on, that’s what I’m scared of.”  “Stress, as that sensation of not having enough time to do this, not knowing when you’re going to be able to do things.”  “I have rules, and norms in the house, so she knows how far she can go. I talk to her.”  “Say this week, her teacher came and said that he was misbehaving, so I talked to him and told him that he must respect his teacher and his mom.”  “Since they’re children, they don’t understand the consequences”  “She plays and then we both pick up, I tell her, let’s pick them up.”  “Last night I told her we had to go to the doctor. So, I explained what the doctor was going to do to her. That’s what I always do.”  “How do I want to teach her? Well, talking to her more every day, explaining to her how things are, what is good and what is bad. What is the right path, and what is the wrong path.”  “We celebrate him a lot, because they say he is taking too long to speak. So right now, we are doing colors. He sees something blue, and he starts saying bl-, bl-, that means blue. So right now, we are praising him for that, so he feels safer to start saying things.”    “We first see what’s right and what’s wrong, what we want to change or what we want to achieve for our daughters’ development and then we make decisions.”  “Say I didn’t like how he brought something up. But in front of the kids, we never fight, it’s after that I can go tell him I don’t agree.”  “There’s a lot of people that help you, for example, my brother, my brother-in-law, or even my wife. I’m not carrying all the weight myself, I call more people, and we manage it together.”  “So when he’s here, he pampers her too much. So then when I go tell her she did something wrong, he tells me not to nag her, to be more patient. And that stresses me.”  “I want her to learn to trust me, to tell me anything, any time, under any circumstance.”  “I always talk to him, and tell him that anything that happens, he can count on his dad, his mom and his brothers”  “If he knows I’m going to get really angry because he took a toy to school, and I’m going to punish him, then he won’t tell me. So, I have to try and talk to them”  “Reading books, and tutorials, I like seeking ways and resources, on how to help children.”  “I say it’s better to talk to someone who knows, not your friend (…) in such cases you need professional help”  “Doing chores while with them.”  “I leave her with my mom, and since I don´t work all day, I work 3 or 4 hours, and then I come back and pick her up.”  “I’ve taught her to play “frozen”, so I say “frozen” and she stays still. And then I tell her “You´re frozen and I´m going to change your diaper”.”  “Her dad got her one (a phone) that sings, so she plays with that.”  “Maybe I don’t have the knowledge, but when the challenge arises, you come up with something.”  “Nobody gave me a script on how to be a dad. There is no manual.”  “My mom spends more time with the child than I do.”  “We´ve been separated for a year and he has only had the boy two weekends.”  “I´m Venezuelan (…) I felt alone, because I don’t have my mom or my sisters´ support.”  “Thanks to God my husband has always been here, he always gives me energy, he tells me: we can do this, we´re going to push through.”  “My mom has always been loving and caring, so we´re like that, and I want my daughter to have that same upbringing.”  “I want to be different than my mom. With her, it was always about hitting us. I don’t want to be that way.”  “You knew that if you did something wrong, then your dad would hit you, so you knew you had to do it well.”  “Each one should educate their children how they want.”  “You have to make them respect you now, because later it becomes harder.”  “Only educating children through dialogue, through respect regarding what’s right and what’s wrong, by explaining things to them”  “I try to stay very calm, because I understand that they’re just children.”  “So he can listen and start understanding, and he will keep growing on his time.”  “I understand that he’s still small, and little by little he will learn” |
