# Supplementary material for: Social and emotional skills for nurturing parenting: A qualitative examination
Source: PLoS One. 2026 Aug 20;21(8):e0355716. doi: 10.1371/journal.pone.0355716 (PMC13492804; doi:10.1371/journal.pone.0355716)
Supplement: Supplementary file 7 [file pone.0355716.s007.docx]

**S7 File.** Inclusivity in Global Research Checklist

Inclusivity in global research

**Ethical considerations, permits and authorship**

*This section is applicable to all research types.*

Provide details as to who granted permissions and/or consent for the study to take place in the Methods section of your manuscript. This should include the names of **all** ethics boards, governmental organizations, community leaders or other bodies that provided approval for the study. If individuals provided approval refer to these people by their role or title but do not list their name(s).

If there were any deviations from the study protocol after approval was obtained please provide details of these changes in the Methods section of your manuscript.

Did this study involve local collaborators that are residents of the country where the research was conducted or members of the community studied? If you do not have any authors from said communities, please provide an explanation for this below.

Yes. The majority of the research team are Colombian nationals with direct professional and/or lived experience in the communities studied. This includes Luciana Beccassino, Juliana Borbón, Jorge Cuartas, Catalina Rey-Guerra, María Alejandra Gutierrez-Torres, and Melissa Guerra — all Colombian researchers affiliated with Fundación Apapacho, a Colombia-based organization. As detailed in the Researcher Positionality statements (Table S2), several team members have direct professional experience working with families in vulnerable communities in Colombia, and some have personal or family history related to the socioeconomic and conflict-related contexts discussed in the study. The study was also conducted in a research-policy partnership with the Instituto Colombiano de Bienestar Familiar (ICBF), which facilitated participant recruitment and shaped elements of the study design based on institutional priorities.

Everyone listed as an author should meet PLOS’ criteria for authorship and all individuals who meet these criteria should be included in the author byline, rather than the acknowledgements. For further information please see the journal’s Authorship Policy.

**Human subjects research (e.g. health research, medical research, cross-cultural psychology)**

Did you obtain written informed consent from a representative of the local community or region before the research took place? How did you establish who speaks for the community? Details of written informed consent obtained from study participants should be reported separately in the Methods section of your manuscript.

Written/oral informed consent was obtained directly from each individual participant, not from a single community representative, since the study targeted individual caregivers accessing public social services rather than a bounded self-governing community. Institutional-level access was authorized by ICBF, through its service units in the selected localities. Ethical approval was obtained from the Harvard University IRB (protocol HRP-503), and oral consent was obtained from all participants.

How did members of the local community provide input on the aims of the research investigation, its methodology, and its anticipated outcome(s)?

Both study instruments were piloted with caregivers outside the final participant pool prior to data collection to ensure appropriateness and cultural relevance. Study design elements were shaped in direct consultation with ICBF, reflecting the institution's frontline knowledge of community needs.

When engaging with the local community, how did you ensure that the informed consent documents and other materials could be understood by local stakeholders?

Will the findings of the research be made available in an understandable format to stakeholders in the community where the study was conducted (e.g. via a presentation, summary report, copies of publications, etc.)? Please provide details of how this will be achieved.

A formal presentation of findings directly to individual participants was not conducted, due to logistical constraints inherent to the study's design (e.g., participants were reached via ICBF service units across dispersed localities, and follow-up contact was not part of the approved protocol). However, findings were shared directly with the Instituto Colombiano de Bienestar Familiar (ICBF), the governmental partner institution through which participants were recruited. These findings directly informed the design of Apapacho, a violence-prevention parenting program that is currently being scaled and adopted through Colombian government programming. In this way, results are reaching and benefiting the broader community of caregivers served by ICBF in an applied, actionable format, even though individual participants were not directly presented with a summary report.

**Non-human subjects research using specimens/ animals collected as part of the study, or those housed in archival collections. Examples include archaeology, paleontology, botany and zoology.**

Did the permission you obtained from a local authority to perform the study include an agreement on access to outputs and benefit sharing? This may include procedures to enable fair distribution of the benefits and resources arising from the research performed. Please include any details of Prior Informed Consent and Benefit Sharing Agreements obtained. These may be required by field-specific regulations, for example the Convention on Biological Diversity (CBD) and the associated Nagoya Protocol.

If the material used in your study was imported, please A) provide the year it was imported and B) indicate whether permits were obtained to import/export the materials used, C) provide details of any permits obtained. If this information is not available, please indicate this.

If you used archival specimens, please state how the material used in your study was acquired by the institute it is held in and provide details of any permits obtained for the original excavations/ sample collection. If this information is not available, please indicate this.

How was the potential cultural significance of the materials collected in your study to local communities considered in your research design? Were Indigenous peoples and/or local researchers and institutions involved with archaeological excavations / collection of specimens? If so, please provide a description of their involvement.

If your manuscript includes photographs of human remains please indicate whether authors obtained permission from descendants or affiliated cultural communities to do so.
