# Supplementary material for: Social and emotional skills for nurturing parenting: A qualitative examination
Source: PLoS One. 2026 Aug 20;21(8):e0355716. doi: 10.1371/journal.pone.0355716 (PMC13492804; doi:10.1371/journal.pone.0355716)
Supplement: Supplementary file 8 [file pone.0355716.s008.docx]

**PONE-D-25-63685 - Social and Emotional Skills for Nurturing Parenting: A Qualitative Examination**

Thank you for the opportunity to review this thoughtful qualitative manuscript. I appreciate the authors’ efforts with an important and underexplored topic. The study has clear potential to contribute meaningful insights to the field. Few comments that can help refine the manuscript further:

1. The abstract is clear and informative; however, there is a substantial portion only on the background information. The authors may wish to streamline the introductory statements and rather highlight the novel contribution of the study viz. parents’ own conceptualizations of relevant social and emotional skills in an LMIC context. Additionally, the description of the two-phase qualitative design could be made slightly more specific to improve clarity.
2. The manuscript alternates between the terms “social and emotional skills” and “social-emotional competence.” The authors may consider standardizing terminology throughout for consistency.
3. Minor wording issue: “ensure interventions are perceived as cultural relevant” should be revised to “culturally relevant.”
4. While the authors aim to assess nurturing parenting, the concept is not explicitly defined. Given that understandings of nurturing parenting may be subjective and context-dependent, providing a clear (even if broad) working definition would improve conceptual clarity for readers.
5. The positionality statement is well-written but could be further elaborated. The authors may consider reflecting on prior experiences working with caregivers, as well as their own caregiving experiences (if applicable), and how these may have influenced data interpretation. Explicitly describing strategies used to mitigate potential bias would strengthen reflexivity.
6. The discussion of multiple social and emotional skills frameworks (e.g., CASEL, BESSI, Jones et al.) is thoughtful. Including a brief description of the process through which the three domains were selected or finalized would enhance transparency.
7. The authors should elaborate on how thematic saturation was assessed and reached in each phase of the study.
8. The data analysis section would benefit from additional detail, particularly regarding whether the analytic process was iterative and which members of the research team were involved at each stage.
9. The coding tree (Figure 1) is well aligned with the narrative description and enhances interpretability. The Results section - there are instances where interpretive statements appear. Would suggest to have a clearer separation between Results and Discussion.
10. Some longer paragraphs in the Results and Discussion sections could be broken up to improve readability and flow.
