# Supplementary material for: Social and emotional skills for nurturing parenting: A qualitative examination
Source: PLoS One. 2026 Aug 20;21(8):e0355716. doi: 10.1371/journal.pone.0355716 (PMC13492804; doi:10.1371/journal.pone.0355716)
Supplement: Supplementary file 9 [file pone.0355716.s010.docx]

**Editor**

1. *Your paper underwent review by two experts in the field. I have also carefully reviewed the manuscript. The manuscript examined an under-researched area on parent’s perceptions of important social-emotional skills needed for caregiving, a topic of relevance and needed to further strengthen parenting interventions in LMICs.*

*I share the generally positive assessments of Reviewers 1 and 2 that this work has strong potential for publication in PLOS ONE. At the same time, both reviewers raise important points that if addressed, would substantially strengthen the manuscript. For this reason, I would like to encourage you to revise and resubmit the paper for further consideration in PLOS ONE.*

*Below, I summarize key themes from the reviews and my own assessment, organized by section. Overall, the manuscript shows promise, but the revision will need to focus especially on substantially strengthening the methodological reporting and the discussion, as these are the areas most critical to improving the paper.*

**Response:** Thank you very much for the comments and the opportunity to revise our manuscript for publication in PLOS ONE. We have used the editor’s and reviewers’ thoughtful and constructive feedback to make substantial edits to the manuscript, which we believe have greatly improved the paper. We also appreciate your clear guidance on the key points to address, all of which we have incorporated into our revisions. Further updates to the manuscript are detailed below.

2. *Background, framing, and context: The introduction provides a solid foundation but would benefit from stronger engagement with literature, where available, on assumptions embedded in parenting programs and on similar analyses in LMICs. In particular, it would help to situate the study within broader debates about whether parenting interventions are informed by parents’ existing practices and knowledge, or introduced without sufficiently understanding what parents already do and know. Given the proliferation of parenting programs in LMICs, this broader framing would clarify the contribution of the study. Reviewer 2 also notes that “nurturing parenting,” although central to the manuscript, is not explicitly defined; clearer conceptual and theoretical grounding would strengthen the framing. Pages 5–8 also offer room to engage more explicitly with literature, or the lack thereof, on the relationships among poverty and hardship, parental socio-emotional skills, and children’s socio-emotional development in LMIC contexts, ideally including settings comparable to Colombia.*

**Response:** We thank the Editor and Reviewer 2 for these helpful suggestions, which have strengthened the framing of the study. We have revised the Introduction and conceptual framework in three ways. First, we now situate the study within current debates about parenting programs in LMICs. We note that most evidence-based programs were developed in high-income countries and later transferred to LMIC settings, and we draw on recent literature to argue that interventions are more likely to be acceptable and sustainable when they are grounded in local caregiving values and knowledge from the outset (Baumann et al., 2019; Mejia et al., 2015, 2017; World Health Organization, 2023). We frame our analysis of parents' own conceptualizations as a necessary first step toward this goal, and connect it to broader concerns about the underrepresentation of LMIC populations in developmental research (Draper et al., 2022; Jeong et al., 2021). This text can be found on p.3. Second, in response to Reviewer 2, we now provide an explicit working definition of nurturing parenting (p.4). We clarify that we use the term to refer to the relational dimension of caregiving, namely warm, responsive, and emotionally supportive parent-child interactions that protect children from harsh treatment and support their social and emotional development. We ground this definition in how caregiving practices are framed in Colombia's national technical guidelines for the prevention of violence against young children (Gobierno de Colombia, 2023), keeping the conceptual framing close to the setting and the families our study describes. Third, we have also strengthened our engagement with the literature on the relationships among poverty, hardship, parents' social and emotional functioning, and children's development (p.8). We now make explicit that a substantial body of international evidence, increasingly including Colombia, links poverty, violence, and chronic hardship to parents' psychological functioning and, through it, to the development and use of the social and emotional skills that nurturing parenting requires, with downstream consequences for children's development (Masarik & Conger, 2017; Zietz et al., 2022; Cuartas, Grogan-Kaylor, et al., 2019; Sánchez-Ariza et al., 2022).

3. *Research questions: Reviewer 1 encourages clearer identification of the qualitative approach guiding the study and suggests formulating the questions in a more interpretive way, moving beyond description toward understanding why parents hold particular views. Reviewer 2 also asks for clarification of how the three focal domains were selected.*

**Response:** Thank you for your comment! We have revised the full text and made the clarification that the qualitative approach for this study was descriptive. The adjustment can be seen in the abstract, as well as p.9 (“*This study aims to explore the social and emotional skills that parents in Colombia perceive as important for providing nurturing parenting through a descriptive qualitative approach*”). The research questions reflect the descriptive approach and focus on parents' perspectives and experiences. We have also clarified how the three focal domains were constructed, building on the researched frameworks and based on the data on p.6: “*These frameworks provided an initial lens for the first stage data analysis in phase 1, and subsequent development of the questionnaire for phase 2 of the study. The final categories responded to parents´descriptions and perspectives as reported in both phases 1 and 2*.” as well as P.16 (“*Three central domains were defined by grouping the social and emotional skills parents identified as relevant to child-rearing (i.e. emotional skills, social skills and adaptability skills).. These domains are a result of a deductive-inductive approach, in which theoretical frameworks informed the collection and initial data analysis, yet the final categories responded to participating parents´perspectives and experiences. While there is a general correspondence between the proposed domains and different frameworks, these were defined not to respond to a specific theoretical model, but to be a reflection of this study´s participants´ lived experiences.*”).

4. *Sample selection: The reviewers request more detail on the random selection procedures. It would also be useful to report the specific indicators used to characterize the selected neighborhoods within the broader city context. In addition, please resolve the discrepancy between the reported focus group participation (2 × 18 = 36) and the stated sample size of 38.*

**Response:** Thank you for your thoughtful comment! We have specified on p.11 the inclusion criteria that all participants met (*“To meet criteria to be beneficiaries, families have to be classified in the System of Identification of Potential Beneficiaries (SISBEN IV) as: living in extreme poverty, moderate poverty or vulnerable to falling into poverty (Instituto Colombiano de Bienestar Familiar, 2021*”). The vulnerability indicators of the selected localities are mentioned afterwards. We have also clarified on p.12 that the random selection of UDSs was carried out by the ICBF. We also specified in p.12 that sample size for phase 1 (*n* = 38) corresponds to a total of 18 focus group participants and 20 individual interviews (The 38 participants were divided into two focus groups with a total of 18 participants (N = 18) and 20 interviews.)

5. *Process and methods: Both reviewers agree that this section requires substantial strengthening. In particular, the manuscript should provide fuller information on interviewer training; procedures to ensure confidentiality and privacy given the sensitivity of the topics; checks for transcription accuracy; coder training; the extent of cross-coding; procedures related to saturation and triangulation; and how divergent or minority perspectives were handled analytically. Please also indicate whether findings were shared with participants or otherwise validated after analysis. In addition, the ethics review currently references only a U.S. institution; please clarify whether local ethical review was sought and, if not, explain why. It would also be helpful to report the number of families reached through the ICBF programs, the number who agreed to participate in Phase 2, and whether the piloting group was similar to the final sample.*

**Response:** We thank the editor and both reviewers for these detailed suggestions, which have substantially strengthened the Method section. We have revised the Measures and Procedures and Data Analysis subsections to address each point, as detailed below.

*Interviewer training* (p.10)*.* All interviews and focus groups were conducted by members of the research team with prior training and experience in qualitative research, all of whom had completed the CITI Program human-subjects research certification (documented in our IRB application). Details on each interviewer's credentials, occupation, gender, and experience are provided in Table S2.

*Confidentiality and privacy* (p.14)*.* Given the sensitivity of the topics, oral informed consent was obtained before each interview and focus group. Telephone interviews were conducted individually, allowing participants to choose a private setting, and focus groups were held at ICBF service units with no non-participants present. Audio recordings and transcripts were stored on password-protected only to the research team, and all transcripts were anonymized. In accordance with the approved IRB protocol, procedures were in place to respond to any participant distress, whereby interviewers would refer participants to support through the established channels of the corresponding ICBF service unit. No such situations arose during data collection.

*Transcription accuracy* (p.14)*.* All interviews and focus groups were transcribed verbatim in Spanish by a native Spanish speaker. To ensure accuracy, all transcripts underwent a double-check process in which they were compared against the original audio recordings and any discrepancies were corrected prior to coding.

*Coder training and cross-coding* (p.15)*.* Coding was conducted by four researchers who received training from a coding team member with extensive qualitative research experience; this team member also reviewed the initial rounds of coding. The four researchers first jointly developed the codebook. To assess consistency, all four independently coded a common section of the data and, given the high level of agreement observed, the remaining transcripts were then divided among them. Coding decisions and discrepancies were resolved through discussion in regular meetings involving all four coders, and these discussions were used to define each code with concrete examples in the codebook. Thus, although not every transcript was coded by two coders, the codebook was developed collaboratively and refined through team consensus.

*Saturation* (p.15)*.* *W*e have added information on data saturation to the Data Analysis section. Phase 1 was part of a broader study with a different focus and was not designed to assess saturation for the present research questions. In Phase 2, designed specifically to explore parents' social and emotional skills, we considered that data saturation was reached, understood as the point at which researchers begin to hear repeated accounts, indicating a sufficient number of participants for a qualitative study (Saunders et al., 2018).

*Triangulation and divergent perspectives* (p.15)*.* We triangulated across individual interviews and focus groups. The codebook captured the social and emotional skills and the risk and protective factors parents identified; divergent and minority perspectives were retained throughout analysis and are reported narratively in the Results.

*Respondent validation* (p.16)*.* Findings were not returned to participants for validation. This was not feasible because interviews were conducted by telephone with participants drawn from different service units and localities, with contact mediated by the ICBF on a single occasion. However, the findings were presented in full to the ICBF to inform the Apapacho parenting program and the institution's broader operations and programs.

*Ethical review* (p.14)*.* Ethical approval was obtained from the Institutional Review Board at Harvard University. In addition, the study underwent a technical approval process by the ICBF, the public agency responsible for child and family policy in Colombia and our partner institution, which was required to access and invite participating families.

*Recruitment numbers and piloting* (p.12)*.* In Phase 1, 473 caregivers were registered and 38 were randomly selected. In Phase 2, the ICBF contacted 28 caregivers to inform them about the study of whom 16 agreed to participate. The piloting groups in both phases were comparable to the final samples, as pilot participants were caregivers drawn from the same ICBF programs and comparable localities who met the same vulnerability criteria.

6. *Results:* *Both reviewers suggest that the analysis should move further beyond description toward deeper interpretation. Greater triangulation across parents’ backgrounds, themes, and domains would strengthen the analysis, and there are already promising examples of this on page 23 that could be extended elsewhere. Examining intersections among SEL domains, between these domains and relevant risk/protective factors, and, where relevant, by child age, could further enrich the findings. Unlike Reviewer 1, I find the inclusion of Ns helpful, as it gives a sense of which perspectives are more common and which are more contrasting. To preserve linguistic and cultural nuance, I also encourage including selected original Spanish excerpts alongside their translations*.

**Response:** We thank you for these suggestions. We have made the clarification that the approach for this study was descriptive (p.9). We have also revised the results section and added insights on the connections between the different social and emotional skills, as well as their relevance for nurturing care. For example, on p.17 we have added: “For these parents, accompanying their children's emotions was a part of helping them solve problems or complete new tasks.” , on p.20: “Furthermore, oftentimes the emotions identified by parents (i.e. anger, stress, fear) acted as mediators on how they managed caregiving, as for example in the case of anger or fear and the use of corporal punishment.” and on p.22: "Parents'desire for building trust with their children acted as motivation for self-regulating and communicating, instead of reacting out of intense emotions or recurring to corporal punishment.” Other relevant insights were added to the other sections of the results. Finally, in line with your comment, we have included the original Spanish excerpts alongside their translations in Table S6.

7. *Discussion: This section should be substantially strengthened. In particular, the discussion needs to engage more explicitly with relevant conceptual frameworks, connect the findings more clearly to existing work in the region, and reflect more directly on how the findings inform assumptions or gaps in current parenting programs. A more analytically integrated discussion, building on stronger triangulation in the results, would significantly improve the manuscript.*

**Response:** We thank the editor for these suggestions, which have substantially strengthened the Discussion. We have made the following revisions.

First, to engage more explicitly with conceptual frameworks, we added a paragraph that maps our three domains onto the social and emotional learning frameworks introduced earlier (CASEL; Jones et al., 2016; the BESSI, Soto et al., 2022) and interprets them through the bioecological model (Bronfenbrenner & Morris, 2007). We note that parents foregrounded adaptive competencies such as managing multiple responsibilities and self-efficacy, that these frameworks tend to underweight and that may be especially salient under economic and social adversity (p.28)*.*

Second, to connect the findings more clearly to existing work in the region, we added a paragraph situating our study within the regional and broader LMIC evidence base. We highlight that parenting interventions in LMICs have shown weaker and less consistent effects on social-emotional than on cognitive, language, and motor outcomes (Zhang et al., 2021), that few programs are locally developed or grounded in parents' own perspectives (Bosqui et al., 2024), and we connect our findings on shared and extended caregiving to work with Latin American families (Parra-Cardona et al., 2018; Syed et al., 2026), alongside the Colombian evidence already cited (p.30)*.*

Third, to reflect more directly on how the findings inform current parenting programs, we added a paragraph to the Implications section illustrating how the skills parents identified informed the design of the Apapacho parenting program, showing how grounding intervention content and recruitment in caregivers' own priorities can enhance cultural relevance and acceptability (p.30)*.*

Finally, to make the discussion more analytically integrated, we revised the emotion-regulation paragraph to frame emotion regulation as a cross-cutting competency that, in parents' accounts, underpinned the social and adaptability skills they valued, rather than treating the three domains in isolation (p.28)*.* We have also clarified that, consistent with our descriptive design, this integrative interpretation is developed at the level of the Discussion, while the Results remain descriptive.

8. *Minor points: Please review the manuscript for statements that require citation (for example, the end of the second paragraph on page 7). In Table 1, please also clarify whether “graduate degree” refers to university education or high school completion.*

**Response:** In response to your comment we have revised all citations, and added the corresponding citation to the statement at the end of the second paragraph on P.9. We have also specified in Table 1 that "Graduate's degree“ refers to university education.

**Reviewer #1**

9. *The authors identify an under-researched area on parent’s perceptions of important social-emotional skills needed for caregiving. The introduction and review build a clear justification for the research questions and identifies key frameworks to guide the study. However, Methods can be elaborated in places. Importantly, the Results can be strengthened with further analysis.*

**Response:** Thank you for the careful review and constructive feedback.

10. *Methods. Present Study: What qualitative research design underpins the approach to qualitative interviewing and analysis (e.g., phemenological, grounded theory)? If none of these were identified, it may be useful to explicitly state this was a descriptive qualitative study.*

**Response:** Thank you for your comment! We have explicitly stated on p.9 that this was a descriptive qualitative study.

11. *Present Study: The research questions could be strengthened to reflect the qualitative approach undertaken. At present, the questions are framed to generate a descriptive list of responses, while qualitative research aims to understand the reasons behind the factors identified (i.e., the how and why). For example, What social and emotional skills to parents living in Colombia perceive as important for providing nurturing parenting, and why?*

**Response:** After revising your comments, we have explicitly stated that this was a descriptive qualitative study on p.9 In line with this approach, our study aimed to explore parents'own accounts of their perspectives and experiences around socioemotional skills. Thus, we have decided to maintain our questions focused on the “what”.

12. *Participant Selection: Was the random selection undertaken by the research team or by the ICBF? Can authors explain why random selection was employed over a more purposeful approach to participant selection and whether the authors believe the random selection permitted a representation of viewpoints of participants in the ICBF programme? It is important to describe this given the value of representation of views in qualitative approaches over generalizability (more important for quantitative approaches).*

**Response:** Thank you for your comment. We have specified on p.12 that the random selection of service units was done by the ICBF. This approach was chosen given that the study took place within the context of an alliance between the research team and the ICBF and one of the aims was to give useful insights that the agency could use to adjust their programs to the needs and characteristics of families, as mentioned on p.12 Therefore, random selection within the selected localities was considered to be an approach that provided a representation of viewpoints of participants in the ICBF programme, considering their shared characteristics mentioned on p.13.

13. *Data Collection: What was the interviewing experience and/or training received y the interview team? How many people were typically included in focus groups? Given the authors mention sensitivity of some topics, what attention was given to privacy during interviewing?*

**Response:** Thank you for these questions. Two of the three points are addressed through the revisions we made in response to the editor's comment on Process and Methods (Comment 5). Specifically, we added that all interviewers were members of the research team with prior training and experience in qualitative research who had completed the CITI Program human-subjects certification, with further detail on each interviewer's credentials and experience in Table S2 (p.10). We also added a description of the measures taken to protect participants' privacy and confidentiality given the sensitivity of the topics, including conducting telephone interviews individually so participants could respond from a private setting, holding focus groups without non-participants present, anonymizing transcripts, securely storing recordings, and following an IRB-approved protocol for participant distress (p.14). In addition, we have now specified that the average number of participants per focus group was 9 (p.12).

14. *Data Analysis: Were there any quality assurance checks for accuracy of transcription?*

**Response:** We thank the reviewer for this question. As noted in our response to the editor's comment on Process and Methods (Comment 5), we have added a quality-assurance check for transcription accuracy to the Data Analysis section: all interviews and focus groups were transcribed verbatim in Spanish by a native Spanish speaker, and all transcripts were then double-checked against the original audio recordings, with any discrepancies corrected prior to coding (p.14).

15. *Data Analysis: Can authors elaborate on triangulation of findings (e.g., between individual and group interviewees, or for example, by parent gender) and the extent to which patterns of deviation alongside common ideas were examined in the analysis process?*

**Response:** Thank you for your comment! We have expanded on the triangulation between individual and group interviews on p.13 with the following: *“Individual interviews provided an in-depth exploration of personal experiences, whereas focus groups facilitated the emergence of additional perspectives through interaction among participants and the creation of a context that can encourage sharing similar or differing views . (Lambert & Loiselle, 2008).”* Given that the focus groups took place in the first phase of the study, the second phase of interviews allowed for a deeper exploration of themes in connection with participant´s personal experiences, thus enriching the analysis.

16. *Data Analysis: Was any respondent validation undertaken? Were preliminary findings shared with parents (or a representative group of parents) to explore their perspectives on interpretation?*

**Response:** We thank the reviewer for this question. As described in our response to the editor's comment on Process and Methods (Comment 5), no respondent validation was undertaken, and we have clarified this in the manuscript. Returning preliminary findings to participants was not feasible because interviews were conducted by telephone with participants drawn from different service units and localities, and contact was mediated by the ICBF on a single occasion. However, the findings were presented in full to the ICBF to inform the Apapacho parenting program and the institution's broader programming (p.16).

17. *Data Analysis: To what extent was saturation of data achieved in phase 1 and phase 2? If this was done, how was saturation defined and what codes or themes could be explored further?*

**Response:** Thank you for this comment. Phase 1 was part of a broader study and was therefore not designed to evaluate saturation in relation to the present study's research questions. In Phase 2, we considered that data saturation was reached, understood as the point at which researchers begin to hear repeated accounts, signaling a sufficient number of participants for a qualitative study (Bek-Pedersen & Montgomery, 2006). The two-phase design also allowed us to deepen the exploration of the themes identified in Phase 1 through more targeted questioning in Phase 2. We have clarified this in the Data Analysis section (p.15).

18. *Results. Figure 1: It is unclear whether the n matters. It may be sufficient to indicate if an idea was common or less commonly expressed; however, both will matter and have value in qualitative research where the breadth of perspectives is of importance. I suggest removing the n in the figure and text.*

**Response:** Thank you for your comment! After revising the suggestions done by both reviewers, we´ve opted for maintaining the n, as recommended by one of the reviewers. Although both common and less commonly expressed ideas were included, we consider the inclusion of Ns can give the reader an idea of which perspectives were more common and which were more contrasting.

19. *Presentation of Findings: Overall, the Results read like a descriptive list with little analysis that offers insight into why parents believe the specific social and emotional skills identified are important in the provision of nurturing care, or influential in their children’s development? It would also be useful to see where findings may have varied, or distinct ideas expressed in interviews compared to the focus groups. At the present, the analysis is limited to the descriptive level. Overall, it will be useful to see a richer analysis.*

**Response:** Thank you for your thoughtful comment. We have revised the results section and added insights on the connections between the different social and emotional skills, as well as their relevance for nurturing care. For example, on p.17 we have added: “For these parents, accompanying their children's emotions was a part of helping them solve problems or complete new tasks.” , on p.20: “Furthermore, oftentimes the emotions identified by parents (i.e. anger, stress, fear) acted as mediators on how they managed caregiving, as for example in the case of anger or fear and the use of corporal punishment.” and on p.22: "Parents' desire for building trust with their children acted as motivation for self-regulating and communicating, instead of reacting out of intense emotions or recurring to corporal punishment.” Other relevant insights were added to the other sections of the results.

20. *Discussion. Limitation: Can authors comment on saturation of findings.*

**Response:** We thank the reviewer for this suggestion. We have added a comment on saturation to the Limitations section (p.32). While we considered that data saturation was reached in Phase 2 (Saunders et al., 2018), Phase 1 was part of a broader study and was not designed to assess saturation for the present research questions. This framing is consistent with our response to Comment 17 and with the clarification added to the Data Analysis section.

**Reviewer #2**

21. *Thank you for the opportunity to review this thoughtful qualitative manuscript. I appreciate the authors’ efforts with an important and underexplored topic. The study has clear potential to contribute meaningful insights to the field. Few comments that can help refine the manuscript further:*

**Response:** Thank you very much for the helpful comments

22. *The abstract is clear and informative; however, there is a substantial portion only on the background information. The authors may wish to streamline the introductory statements and rather highlight the novel contribution of the study viz. parents’ own conceptualizations of relevant social and emotional skills in an LMIC context. Additionally, the description of the two-phase qualitative design could be made slightly more specific to improve clarity.*

**Response:** We thank the reviewer for this helpful suggestion! We have revised the abstract to streamline the background and foreground of the study's novel contribution: parents' own conceptualizations of the social and emotional skills relevant to parenting in an LMIC context. We have also made the two-phase design more specific, clarifying that the first phase combined focus groups and individual interviews and the second phase consisted of targeted individual interviews focused on social and emotional skills (p.2).

23. *The manuscript alternates between the terms “social and emotional skills” and “social-emotional competence.” The authors may consider standardizing terminology throughout for consistency.*

**Response:** Thank you for this suggestion! We have standardized the terminology throughout the manuscript and now consistently use "social and emotional skills," replacing instances of "social-emotional competence".

24. *Minor wording issue: “ensure interventions are perceived as cultural relevant” should be revised to “culturally relevant.”*

**Response:** Thank you for your comment! The wording was adjusted to be “culturally relevant” on p.4.

25. *While the authors aim to assess nurturing parenting, the concept is not explicitly defined. Given that understandings of nurturing parenting may be subjective and context-dependent, providing a clear (even if broad) working definition would improve conceptual clarity for readers.*

**Response:** Thank you for this helpful point. We agree that nurturing parenting should be defined explicitly. We have added a working definition to the Conceptual Framework section, clarifying that we use nurturing parenting to refer to the relational dimension of caregiving, namely the warm, responsive, and emotionally supportive interactions through which parents build secure bonds with their children, respond to their needs, and protect them from harsh or violent treatment. We further specify that our focus is on the parenting skills and interactions that support children's social and emotional development, rather than on other domains of care such as health or nutrition. Recognizing that understandings of nurturing parenting are context-dependent, we ground our definition in Colombia's national technical guidelines for the prevention of violence against young children (Gobierno de Colombia, 2023) (p.4).

26. *The positionality statement is well-written but could be further elaborated. The authors may consider reflecting on prior experiences working with caregivers, as well as their own caregiving experiences (if applicable), and how these may have influenced data interpretation. Explicitly describing strategies used to mitigate potential bias would strengthen reflexivity.*

**Response:** Thank you for this helpful suggestion! We have elaborated the Researcher Positionality section (p.11) and the Table S2 (Supporting Information) to include each researcher's prior experience working with caregivers (ranging from approximately 5 to 25 years across the team) and, where applicable, their own caregiving experiences (two team members are mothers), noting how these may shape interpretation. We have also made explicit the strategies used to mitigate potential bias such as reflexive discussion among members with differing backgrounds, collaborative coding by multiple coders, and anchoring analytic decisions in participants' own words.

27. *The discussion of multiple social and emotional skills frameworks (e.g., CASEL, BESSI, Jones et al.) is thoughtful. Including a brief description of the process through which the three domains were selected or finalized would enhance transparency.*

**Response:** Thank you for your comment. We have clarified how the three focal domains were constructed and finalized, building on the researched frameworks and based on the data on p.6: “*These frameworks provided an initial lens for the first stage data analysis in phase 1, and subsequent development of the questionnaire for phase 2 of the study. The final categories responded to parents´descriptions and perspectives as reported in both phases 1 and 2*.” as well as P.16 (“*Three central domains were defined by grouping the social and emotional skills parents identified as relevant to child-rearing (i.e. emotional skills, social skills and adaptability skills).. These domains are a result of a deductive-inductive approach, in which theoretical frameworks informed the collection and initial data analysis, yet the final categories responded to participating parents´perspectives and experiences. While there is a general correspondence between the proposed domains and different frameworks, these were defined not to respond to a specific theoretical model, but to be a reflection of this study´s participants´ lived experiences.*”).

28. *The authors should elaborate on how thematic saturation was assessed and reached in each phase of the study.*

**Response:** Thank you for this question. As noted in our responses to Comments 17 and 20, Phase 1 was part of a broader study and was not designed to assess saturation for the present research questions. In Phase 2, designed specifically to explore parents' social and emotional skills, we considered that data saturation was reached, understood as the point at which researchers begin to hear repeated accounts, indicating a sufficient number of participants for a qualitative study (Saunders et al., 2018). We have clarified this in the Data Analysis section (p.15) and noted it in the Limitations (p.32).

29. *The data analysis section would benefit from additional detail, particularly regarding whether the analytic process was iterative and which members of the research team were involved at each stage.*

**Response:** Thank you for this suggestion! We have expanded the Data Analysis section with additional detail on the analytic process. We clarified that coding was conducted by four researchers following a collaborative process, that all four coders were trained by a team member with extensive qualitative experience who also reviewed their initial coding, and that the team jointly developed the codebook, assessed consistency by independently coding a common subset of the data, and resolved coding decisions and discrepancies through regular team meetings, refining the codebook through consensus. We also specify that the analytic process was iterative across both phases, involving repeated cycles of coding, team discussion, codebook refinement, and re-coding, and we direct readers to Table S2, which details the contributions of each team member at each stage of the study (p.15).

30. *The coding tree (Figure 1) is well aligned with the narrative description and enhances interpretability. The Results section - there are instances where interpretive statements appear. Would suggest to have a clearer separation between Results and Discussion.*

**Response:** Thank you for this helpful observation, and for noting that the coding tree aligns well with the narrative description. We agree that the Results and Discussion should be more clearly delineated, and we have revised the manuscript accordingly.

In doing so, we aimed to balance this suggestion with the editor's and Reviewer 1's request (Comments 6 and 19) for a richer account of the connections among codes, themes, and domains. To reconcile both, we adopted the following distinction: the Results section now presents (a) the codes and themes that emerged from participants' accounts and (b) the connections among them as expressed in the data (e.g., how participants linked their understanding of children's developmental processes to greater patience and emotional regulation), without inferential or theoretical claims. Statements that interpreted or contextualized these findings were located in the Discussion.

31. *Some longer paragraphs in the Results and Discussion sections could be broken up to improve readability and flow.*

**Response:** Thank you for your comment! We have revised the Results and Discussion sections and broken down several paragraphs for a better readability. For example, on p.X we separated in different paragraphs the analysis on how the social and emotional skills identified in our study align with existing literature, and how they align with existing social and emotional learning frameworks, specifically.
