# Supplementary material for: Social and emotional skills for nurturing parenting: A qualitative examination
Source: PLoS One. 2026 Aug 20;21(8):e0355716. doi: 10.1371/journal.pone.0355716 (PMC13492804; doi:10.1371/journal.pone.0355716)
Supplement: Peer review history

# Peer review: Decision Letter 0

**DOI:** 10.1371/journal.pone.0355716.r001

**Authors:** Stefaan Six

22 Mar 2026

Social and Emotional Skills for Nurturing Parenting: A Qualitative Examination

PLOS One

Dear Dr. Cuartas,

Thank you for submitting your manuscript to PLOS ONE. After careful consideration, we feel that it has merit but does not fully meet PLOS ONE’s publication criteria as it currently stands. Therefore, we invite you to submit a revised version of the manuscript that addresses the points raised during the review process.

Please submit your revised manuscript by May 06 2026 11:59PM. If you will need more time than this to complete your revisions, please reply to this message or contact the journal office at plosone@plos.org. When you're ready to submit your revision, log on to https://www.editorialmanager.com/pone/ and select the 'Submissions Needing Revision' folder to locate your manuscript file.

- A letter that responds to each point raised by the academic editor and reviewer(s). You should upload this letter as a separate file labeled 'Response to Reviewers'.
- A marked-up copy of your manuscript that highlights changes made to the original version. You should upload this as a separate file labeled 'Revised Manuscript with Track Changes'.
- An unmarked version of your revised paper without tracked changes. You should upload this as a separate file labeled 'Manuscript'.

If you would like to make changes to your financial disclosure, please include your updated statement in your cover letter. Guidelines for resubmitting your figure files are available below the reviewer comments at the end of this letter.

If applicable, we recommend that you deposit your laboratory protocols in protocols.io to enhance the reproducibility of your results. Protocols.io assigns your protocol its own identifier (DOI) so that it can be cited independently in the future. For instructions see: https://journals.plos.org/plosone/s/submission-guidelines#loc-laboratory-protocols. Additionally, PLOS ONE offers an option for publishing peer-reviewed Lab Protocol articles, which describe protocols hosted on protocols.io. Read more information on sharing protocols at https://plos.org/protocols?utm_medium=editorial-email&utm_source=authorletters&utm_campaign=protocols.

We look forward to receiving your revised manuscript.

Kind regards,

Stefaan Six, Ph.D.

Academic Editor

PLOS One

Journal Requirements:

When submitting your revision, we need you to address these additional requirements.

1.Please ensure that your manuscript meets PLOS ONE's style requirements, including those for file naming. The PLOS ONE style templates can be found at

https://journals.plos.org/plosone/s/file?id=wjVg/PLOSOne_formatting_sample_main_body.pdf and

https://journals.plos.org/plosone/s/file?id=ba62/PLOSOne_formatting_sample_title_authors_affiliations.pdf

2. In the ethics statement in the Methods, you have specified that verbal consent was obtained. Please provide additional details regarding how this consent was documented and witnessed, and state whether this was approved by the IRB.

3. Please include a complete copy of PLOS’ questionnaire on inclusivity in global research in your revised manuscript. Our policy for research in this area aims to improve transparency in the reporting of research performed outside of researchers’ own country or community. The policy applies to researchers who have travelled to a different country to conduct research, research with Indigenous populations or their lands, and research on cultural artefacts. The questionnaire can also be requested at the journal’s discretion for any other submissions, even if these conditions are not met.  Please find more information on the policy and a link to download a blank copy of the questionnaire here: https://journals.plos.org/plosone/s/best-practices-in-research-reporting. Please upload a completed version of your questionnaire as Supporting Information when you resubmit your manuscript.

4. Thank you for stating the following in your Competing Interests section:

“No competing interests to disclose”

Please complete your Competing Interests on the online submission form to state any Competing Interests. If you have no competing interests, please state "The authors have declared that no competing interests exist.", as detailed online in our guide for authors at http://journals.plos.org/plosone/s/submit-now

This information should be included in your cover letter; we will change the online submission form on your behalf.

5. We note that you have indicated that there are restrictions to data sharing for this study. PLOS only allows data to be available upon request if there are legal or ethical restrictions on sharing data publicly. For more information on unacceptable data access restrictions, please see http://journals.plos.org/plosone/s/data-availability#loc-unacceptable-data-access-restrictions.

Before we proceed with your manuscript, please address the following prompts:

a) If there are ethical or legal restrictions on sharing a de-identified data set, please explain them in detail (e.g., data contain potentially identifying or sensitive patient information, data are owned by a third-party organization, etc.) and who has imposed them (e.g., a Research Ethics Committee or Institutional Review Board, etc.). Please also provide contact information for a data access committee, ethics committee, or other institutional body to which data requests may be sent.

b) If there are no restrictions, please upload the minimal anonymized data set necessary to replicate your study findings to a stable, public repository and provide us with the relevant URLs, DOIs, or accession numbers. For a list of recommended repositories, please see

https://journals.plos.org/plosone/s/recommended-repositories. You also have the option of uploading the data as Supporting Information files, but we would recommend depositing data directly to a data repository if possible.

We will update your Data Availability statement on your behalf to reflect the information you provide.

6. Please include your full ethics statement in the ‘Methods’ section of your manuscript file. In your statement, please include the full name of the IRB or ethics committee who approved or waived your study, as well as whether or not you obtained informed written or verbal consent. If consent was waived for your study, please include this information in your statement as well.

7. Please include captions for your Supporting Information files at the end of your manuscript, and update any in-text citations to match accordingly. Please see our Supporting Information guidelines for more information: http://journals.plos.org/plosone/s/supporting-information.

8. If the reviewer comments include a recommendation to cite specific previously published works, please review and evaluate these publications to determine whether they are relevant and should be cited. There is no requirement to cite these works unless the editor has indicated otherwise.

Additional Editor Comments:

Your paper underwent review by two experts in the field. I have also carefully reviewed the manuscript. The manuscript examined an under-researched area on parent’s perceptions of important social-emotional skills needed for caregiving, a topic of relevance and needed to further strengthen parenting interventions in LMICs.

I share the generally positive assessments of Reviewers 1 and 2 that this work has strong potential for publication in PLOS ONE. At the same time, both reviewers raise important points that if addressed, would substantially strengthen the manuscript. For this reason, I would like to encourage you to revise and resubmit the paper for further consideration in PLOS ONE.

Below, I summarize key themes from the reviews and my own assessment, organized by section. Overall, the manuscript shows promise, but the revision will need to focus especially on substantially strengthening the methodological reporting and the discussion, as these are the areas most critical to improving the paper.

**Background, framing, and context:**

The introduction provides a solid foundation but would benefit from stronger engagement with literature, where available, on assumptions embedded in parenting programs and on similar analyses in LMICs. In particular, it would help to situate the study within broader debates about whether parenting interventions are informed by parents’ existing practices and knowledge, or introduced without sufficiently understanding what parents already do and know. Given the proliferation of parenting programs in LMICs, this broader framing would clarify the contribution of the study. Reviewer 2 also notes that “nurturing parenting,” although central to the manuscript, is not explicitly defined; clearer conceptual and theoretical grounding would strengthen the framing. Pages 5–8 also offer room to engage more explicitly with literature, or the lack thereof, on the relationships among poverty and hardship, parental socio-emotional skills, and children’s socio-emotional development in LMIC contexts, ideally including settings comparable to Colombia.

**Research questions:**

Reviewer 1 encourages clearer identification of the qualitative approach guiding the study and suggests formulating the questions in a more interpretive way, moving beyond description toward understanding why parents hold particular views. Reviewer 2 also asks for clarification of how the three focal domains were selected.

**Sample selection:**

The reviewers request more detail on the random selection procedures. It would also be useful to report the specific indicators used to characterize the selected neighborhoods within the broader city context. In addition, please resolve the discrepancy between the reported focus group participation (2 × 18 = 36) and the stated sample size of 38.

**Process and methods:**

Both reviewers agree that this section requires substantial strengthening. In particular, the manuscript should provide fuller information on interviewer training; procedures to ensure confidentiality and privacy given the sensitivity of the topics; checks for transcription accuracy; coder training; the extent of cross-coding; procedures related to saturation and triangulation; and how divergent or minority perspectives were handled analytically. Please also indicate whether findings were shared with participants or otherwise validated after analysis. In addition, the ethics review currently references only a U.S. institution; please clarify whether local ethical review was sought and, if not, explain why. It would also be helpful to report the number of families reached through the ICBF programs, the number who agreed to participate in Phase 2, and whether the piloting group was similar to the final sample.

**Results:**

Both reviewers suggest that the analysis should move further beyond description toward deeper interpretation. Greater triangulation across parents’ backgrounds, themes, and domains would strengthen the analysis, and there are already promising examples of this on page 23 that could be extended elsewhere. Examining intersections among SEL domains, between these domains and relevant risk/protective factors, and, where relevant, by child age, could further enrich the findings. Unlike Reviewer 1, I find the inclusion of Ns helpful, as it gives a sense of which perspectives are more common and which are more contrasting. To preserve linguistic and cultural nuance, I also encourage including selected original Spanish excerpts alongside their translations.

**Discussion:**

This section should be substantially strengthened. In particular, the discussion needs to engage more explicitly with relevant conceptual frameworks, connect the findings more clearly to existing work in the region, and reflect more directly on how the findings inform assumptions or gaps in current parenting programs. A more analytically integrated discussion, building on stronger triangulation in the results, would significantly improve the manuscript.

**Minor points:**

Please review the manuscript for statements that require citation (for example, the end of the second paragraph on page 7). In Table 1, please also clarify whether “graduate degree” refers to university education or high school completion.

I encourage you to address the issues and comments raised in the individual reviews and to submit a revised manuscript. When resubmitting, please include a detailed response letter outlining the changes made and explaining how you addressed (or chose not to address) each reviewer and editor comment. I anticipate sending the revised manuscript back to the original reviewers.

[Note: HTML markup is below. Please do not edit.]

Reviewers' comments:

Reviewer's Responses to Questions

**Comments to the Author**

1. Is the manuscript technically sound, and do the data support the conclusions?

Reviewer #1: Yes

Reviewer #2: Yes

**********

2. Has the statistical analysis been performed appropriately and rigorously?

Reviewer #1: N/A

Reviewer #2: N/A

**********

3. Have the authors made all data underlying the findings in their manuscript fully available?

The PLOS Data policy

Reviewer #1: Yes

Reviewer #2: No

**********

4. Is the manuscript presented in an intelligible fashion and written in standard English?

Reviewer #1: Yes

Reviewer #2: Yes

**********

Reviewer #1: Overview

The authors identify an under-researched area on parent’s perceptions of important social-emotional skills needed for caregiving. The introduction and review build a clear justification for the research questions and identifies key frameworks to guide the study. However, Methods can be elaborated in places. Importantly, the Results can be strengthened with further analysis.

Specific Comments

• Methods

o Present Study: What qualitative research design underpins the approach to qualitative interviewing and analysis (e.g., phemenological, grounded theory)? If none of these were identified, it may be useful to explicitly state this was a descriptive qualitative study.

o Present Study: The research questions could be strengthened to reflect the qualitative approach undertaken. At present, the questions are framed to generate a descriptive list of responses, while qualitative research aims to understand the reasons behind the factors identified (i.e., the how and why). For example, What social and emotional skills to parents living in Colombia perceive as important for providing nurturing parenting, and why?

o Participant Selection: Was the random selection undertaken by the research team or by the ICBF? Can authors explain why random selection was employed over a more purposeful approach to participant selection and whether the authors believe the random selection permitted a representation of viewpoints of participants in the ICBF programme? It is important to describe this given the value of representation of views in qualitative approaches over generalizability (more important for quantitative approaches).

o Data Collection: What was the interviewing experience and/or training received by the interview team? How many people were typically included in focus groups? Given the authors mention sensitivity of some topics, what attention was given to privacy during interviewing?

o Data Analysis: Were there any quality assurance checks for accuracy of transcription?

o Data Analysis: Can authors elaborate on triangulation of findings (e.g., between individual and group interviewees, or for example, by parent gender) and the extent to which patterns of deviation alongside common ideas were examined in the analysis process?

o Data Analysis: Was any respondent validation undertaken? Were preliminary findings shared with parents (or a representative group of parents) to explore their perspectives on interpretation?

o Data Analysis: To what extent was saturation of data achieved in phase 1 and phase 2? If this was done, how was saturation defined and what codes or themes could be explored further?

• Results

o Figure 1: It is unclear whether the n matters. It may be sufficient to indicate if an idea was common or less commonly expressed; however, both will matter and have value in qualitative research where the breadth of perspectives is of importance. I suggest removing the n in the figure and text.

o Presentation of Findings: Overall, the Results read like a descriptive list with little analysis that offers insight into why parents believe the specific social and emotional skills identified are important in the provision of nurturing care, or influential in their children’s development? It would also be useful to see where findings may have varied, or distinct ideas expressed in interviews compared to the focus groups. At the present, the analysis is limited to the descriptive level. Overall, it will be useful to see a richer analysis.

• Discussion

o Limitation: Can authors comment on saturation of findings.

Thank you for the opportunity to review this paper.

Reviewer #2: PONE-D-25-63685 - Social and Emotional Skills for Nurturing Parenting: A Qualitative Examination

Thank you for the opportunity to review this thoughtful qualitative manuscript. I appreciate the authors’ efforts with an important and underexplored topic. The study has clear potential to contribute meaningful insights to the field. Few comments that can help refine the manuscript further:

1. The abstract is clear and informative; however, there is a substantial portion only on the background information. The authors may wish to streamline the introductory statements and rather highlight the novel contribution of the study viz. parents’ own conceptualizations of relevant social and emotional skills in an LMIC context. Additionally, the description of the two-phase qualitative design could be made slightly more specific to improve clarity.

2. The manuscript alternates between the terms “social and emotional skills” and “social-emotional competence.” The authors may consider standardizing terminology throughout for consistency.

3. Minor wording issue: “ensure interventions are perceived as cultural relevant” should be revised to “culturally relevant.”

4. While the authors aim to assess nurturing parenting, the concept is not explicitly defined. Given that understandings of nurturing parenting may be subjective and context-dependent, providing a clear (even if broad) working definition would improve conceptual clarity for readers.

5. The positionality statement is well-written but could be further elaborated. The authors may consider reflecting on prior experiences working with caregivers, as well as their own caregiving experiences (if applicable), and how these may have influenced data interpretation. Explicitly describing strategies used to mitigate potential bias would strengthen reflexivity.

6. The discussion of multiple social and emotional skills frameworks (e.g., CASEL, BESSI, Jones et al.) is thoughtful. Including a brief description of the process through which the three domains were selected or finalized would enhance transparency.

7. The authors should elaborate on how thematic saturation was assessed and reached in each phase of the study.

8. The data analysis section would benefit from additional detail, particularly regarding whether the analytic process was iterative and which members of the research team were involved at each stage.

9. The coding tree (Figure 1) is well aligned with the narrative description and enhances interpretability. The Results section - there are instances where interpretive statements appear. Would suggest to have a clearer separation between Results and Discussion.

10. Some longer paragraphs in the Results and Discussion sections could be broken up to improve readability and flow.

**********

what does this mean?). If published, this will include your full peer review and any attached files.

If you choose “no”, your identity will remain anonymous but your review may still be made public.

**Do you want your identity to be public for this peer review?**  For information about this choice, including consent withdrawal, please see our Privacy Policy

Reviewer #1: No

Reviewer #2: **Yes:** Eunice Lobo

**********

[NOTE: If reviewer comments were submitted as an attachment file, they will be attached to this email and accessible via the submission site. Please log into your account, locate the manuscript record, and check for the action link "View Attachments". If this link does not appear, there are no attachment files.]

To ensure your figures meet our technical requirements, please review our figure guidelines: https://journals.plos.org/plosone/s/figures

You may also use PLOS’s free figure tool, NAAS, to help you prepare publication quality figures: https://journals.plos.org/plosone/s/figures#loc-tools-for-figure-preparation.

NAAS will assess whether your figures meet our technical requirements by comparing each figure against our figure specifications.

Attachment Submitted filename: Review report.docx

# Author comment: Author response to Decision Letter 1

**DOI:** 10.1371/journal.pone.0355716.r002

10 Jul 2026

Editor

1. Your paper underwent review by two experts in the field. I have also carefully reviewed the manuscript. The manuscript examined an under-researched area on parent’s perceptions of important social-emotional skills needed for caregiving, a topic of relevance and needed to further strengthen parenting interventions in LMICs.

I share the generally positive assessments of Reviewers 1 and 2 that this work has strong potential for publication in PLOS ONE. At the same time, both reviewers raise important points that if addressed, would substantially strengthen the manuscript. For this reason, I would like to encourage you to revise and resubmit the paper for further consideration in PLOS ONE.

Below, I summarize key themes from the reviews and my own assessment, organized by section. Overall, the manuscript shows promise, but the revision will need to focus especially on substantially strengthening the methodological reporting and the discussion, as these are the areas most critical to improving the paper.

Response: Thank you very much for the comments and the opportunity to revise our manuscript for publication in PLOS ONE. We have used the editor’s and reviewers’ thoughtful and constructive feedback to make substantial edits to the manuscript, which we believe have greatly improved the paper. We also appreciate your clear guidance on the key points to address, all of which we have incorporated into our revisions. Further updates to the manuscript are detailed below.

2. Background, framing, and context: The introduction provides a solid foundation but would benefit from stronger engagement with literature, where available, on assumptions embedded in parenting programs and on similar analyses in LMICs. In particular, it would help to situate the study within broader debates about whether parenting interventions are informed by parents’ existing practices and knowledge, or introduced without sufficiently understanding what parents already do and know. Given the proliferation of parenting programs in LMICs, this broader framing would clarify the contribution of the study. Reviewer 2 also notes that “nurturing parenting,” although central to the manuscript, is not explicitly defined; clearer conceptual and theoretical grounding would strengthen the framing. Pages 5–8 also offer room to engage more explicitly with literature, or the lack thereof, on the relationships among poverty and hardship, parental socio-emotional skills, and children’s socio-emotional development in LMIC contexts, ideally including settings comparable to Colombia.

Response: We thank the Editor and Reviewer 2 for these helpful suggestions, which have strengthened the framing of the study. We have revised the Introduction and conceptual framework in three ways. First, we now situate the study within current debates about parenting programs in LMICs. We note that most evidence-based programs were developed in high-income countries and later transferred to LMIC settings, and we draw on recent literature to argue that interventions are more likely to be acceptable and sustainable when they are grounded in local caregiving values and knowledge from the outset (Baumann et al., 2019; Mejia et al., 2015, 2017; World Health Organization, 2023). We frame our analysis of parents' own conceptualizations as a necessary first step toward this goal, and connect it to broader concerns about the underrepresentation of LMIC populations in developmental research (Draper et al., 2022; Jeong et al., 2021). This text can be found on p.3. Second, in response to Reviewer 2, we now provide an explicit working definition of nurturing parenting (p.4). We clarify that we use the term to refer to the relational dimension of caregiving, namely warm, responsive, and emotionally supportive parent-child interactions that protect children from harsh treatment and support their social and emotional development. We ground this definition in how caregiving practices are framed in Colombia's national technical guidelines for the prevention of violence against young children (Gobierno de Colombia, 2023), keeping the conceptual framing close to the setting and the families our study describes. Third, we have also strengthened our engagement with the literature on the relationships among poverty, hardship, parents' social and emotional functioning, and children's development (p.8). We now make explicit that a substantial body of international evidence, increasingly including Colombia, links poverty, violence, and chronic hardship to parents' psychological functioning and, through it, to the development and use of the social and emotional skills that nurturing parenting requires, with downstream consequences for children's development (Masarik & Conger, 2017; Zietz et al., 2022; Cuartas, Grogan-Kaylor, et al., 2019; Sánchez-Ariza et al., 2022).

3. Research questions: Reviewer 1 encourages clearer identification of the qualitative approach guiding the study and suggests formulating the questions in a more interpretive way, moving beyond description toward understanding why parents hold particular views. Reviewer 2 also asks for clarification of how the three focal domains were selected.

Response: Thank you for your comment! We have revised the full text and made the clarification that the qualitative approach for this study was descriptive. The adjustment can be seen in the abstract, as well as p.9 (“This study aims to explore the social and emotional skills that parents in Colombia perceive as important for providing nurturing parenting through a descriptive qualitative approach”). The research questions reflect the descriptive approach and focus on parents' perspectives and experiences. We have also clarified how the three focal domains were constructed, building on the researched frameworks and based on the data on p.6: “These frameworks provided an initial lens for the first stage data analysis in phase 1, and subsequent development of the questionnaire for phase 2 of the study. The final categories responded to parents´descriptions and perspectives as reported in both phases 1 and 2.” as well as P.16 (“Three central domains were defined by grouping the social and emotional skills parents identified as relevant to child-rearing (i.e. emotional skills, social skills and adaptability skills).. These domains are a result of a deductive-inductive approach, in which theoretical frameworks informed the collection and initial data analysis, yet the final categories responded to participating parents´perspectives and experiences. While there is a general correspondence between the proposed domains and different frameworks, these were defined not to respond to a specific theoretical model, but to be a reflection of this study´s participants´ lived experiences.”).

4. Sample selection: The reviewers request more detail on the random selection procedures. It would also be useful to report the specific indicators used to characterize the selected neighborhoods within the broader city context. In addition, please resolve the discrepancy between the reported focus group participation (2 × 18 = 36) and the stated sample size of 38.

Response: Thank you for your thoughtful comment! We have specified on p.11 the inclusion criteria that all participants met (“To meet criteria to be beneficiaries, families have to be classified in the System of Identification of Potential Beneficiaries (SISBEN IV) as: living in extreme poverty, moderate poverty or vulnerable to falling into poverty (Instituto Colombiano de Bienestar Familiar, 2021”). The vulnerability indicators of the selected localities are mentioned afterwards. We have also clarified on p.12 that the random selection of UDSs was carried out by the ICBF. We also specified in p.12 that sample size for phase 1 (n = 38) corresponds to a total of 18 focus group participants and 20 individual interviews (The 38 participants were divided into two focus groups with a total of 18 participants (N = 18) and 20 interviews.)

5. Process and methods: Both reviewers agree that this section requires substantial strengthening. In particular, the manuscript should provide fuller information on interviewer training; procedures to ensure confidentiality and privacy given the sensitivity of the topics; checks for transcription accuracy; coder training; the extent of cross-coding; procedures related to saturation and triangulation; and how divergent or minority perspectives were handled analytically. Please also indicate whether findings were shared with participants or otherwise validated after analysis. In addition, the ethics review currently references only a U.S. institution; please clarify whether local ethical review was sought and, if not, explain why. It would also be helpful to report the number of families reached through the ICBF programs, the number who agreed to participate in Phase 2, and whether the piloting group was similar to the final sample.

Response: We thank the editor and both reviewers for these detailed suggestions, which have substantially strengthened the Method section. We have revised the Measures and Procedures and Data Analysis subsections to address each point, as detailed below.

Interviewer training (p.10). All interviews and focus groups were conducted by members of the research team with prior training and experience in qualitative research, all of whom had completed the CITI Program human-subjects research certification (documented in our IRB application). Details on each interviewer's credentials, occupation, gender, and experience are provided in Table S2.

Confidentiality and privacy (p.14). Given the sensitivity of the topics, oral informed consent was obtained before each interview and focus group. Telephone interviews were conducted individually, allowing participants to choose a private setting, and focus groups were held at ICBF service units with no non-participants present. Audio recordings and transcripts were stored on password-protected only to the research team, and all transcripts were anonymized. In accordance with the approved IRB protocol, procedures were in place to respond to any participant distress, whereby interviewers would refer participants to support through the established channels of the corresponding ICBF service unit. No such situations arose during data collection.

Transcription accuracy (p.14). All interviews and focus groups were transcribed verbatim in Spanish by a native Spanish speaker. To ensure accuracy, all transcripts underwent a double-check process in which they were compared against the original audio recordings and any discrepancies were corrected prior to coding.

Coder training and cross-coding (p.15). Coding was conducted by four researchers who received training from a coding team member with extensive qualitative research experience; this team member also reviewed the initial rounds of coding. The four researchers first jointly developed the codebook. To assess consistency, all four independently coded a common section of the data and, given the high level of agreement observed, the remaining transcripts were then divided among them. Coding decisions and discrepancies were resolved through discussion in regular meetings involving all four coders, and these discussions were used to define each code with concrete examples in the codebook. Thus, although not every transcript was coded by two coders, the codebook was developed collaboratively and refined through team consensus.

Saturation (p.15). We have added information on data saturation to the Data Analysis section. Phase 1 was part of a broader study with a different focus and was not designed to assess saturation for the present research questions. In Phase 2, designed specifically to explore parents' social and emotional skills, we considered that data saturation was reached, understood as the point at which researchers begin to hear repeated accounts, indicating a sufficient number of participants for a qualitative study (Saunders et al., 2018).

Triangulation and divergent perspectives (p.15). We triangulated across individual interviews and focus groups. The codebook captured the social and emotional skills and the risk and protective factors parents identified; divergent and minority perspectives were retained throughout analysis and are reported narratively in the Results.

Respondent validation (p.16). Findings were not returned to participants for validation. This was not feasible because interviews were conducted by telephone with participants drawn from different service units and localities, with contact mediated by the ICBF on a single occasion. However, the findings were presented in full to the ICBF to inform the Apapacho parenting program and the institution's broader operations and programs.

Ethical review (p.14). Ethical approval was obtained from the Institutional Review Board at Harvard University. In addition, the study underwent a technical approval process by the ICBF, the public agency responsible for child and family policy in Colombia and our partner institution, which was required to access and invite participating families.

Recruitment numbers and piloting (p.12). In Phase 1, 473 caregivers were registered and 38 were randomly selected. In Phase 2, the ICBF contacted 28 caregivers to inform them about the study of whom 16 agreed to participate. The piloting groups in both phases were comparable to the final samples, as pilot participants were caregivers drawn from the same ICBF programs and comparable localities who met the same vulnerability criteria.

6. Results: Both reviewers suggest that the analysis should move further beyond description toward deeper interpretation. Greater triangulation across parents’ backgrounds, themes, and domains would strengthen the analysis, and there are already promising examples of this on page 23 that could be extended elsewhere. Examining intersections among SEL domains, between these domains and relevant risk/protective factors, and, where relevant, by child age, could further enrich the findings. Unlike Reviewer 1, I find the inclusion of Ns helpful, as it gives a sense of which perspectives are more common and which are more contrasting. To preserve linguistic and cultural nuance, I also encourage including selected original Spanish excerpts alongside their translations.

Response: We thank you for these suggestions. We have made the clarification that the approach for this study was descriptive (p.9). We have also revised the results section and added insights on the connections between the different social and emotional skills, as well as their relevance for nurturing care. For example, on p.17 we have added: “For these parents, accompanying their children's emotions was a part of helping them solve problems or complete new tasks.” , on p.20: “Furthermore, oftentimes the emotions identified by parents (i.e. anger, stress, fear) acted as mediators on how they managed caregiving, as for example in the case of anger or fear and the use of corporal punishment.” and on p.22: "Parents'desire for building trust with their children acted as motivation for self-regulating and communicating, instead of reacting out of intense emotions or recurring to corporal punishment.” Other relevant insights were added to the other sections of the results. Finally, in line with your comment, we have included the original Spanish excerpts alongside their translations in Table S6.

7. Discussion: This section should be substantially strengthened. In particular, the discussion needs to engage more explicitly with relevant conceptual frameworks, connect the findings more clearly to existing work in the region, and reflect more directly on how the findings inform assumptions or gaps in current parenting programs. A more analytically integrated discussion, building on stronger triangulation in the results, would significantly improve the manuscript.

Response: We thank the editor for these suggestions, which have substantially strengthened the Discussion. We have made the following revisions.

First, to engage more explicitly with conceptual frameworks, we added a paragraph that maps our three domains onto the social and emotional learning frameworks introduced earlier (CASEL; Jones et al., 2016; the BESSI, Soto et

Attachment Submitted filename: response to reviewers comments.docx

# Peer review: Decision Letter 1

**DOI:** 10.1371/journal.pone.0355716.r003

**Authors:** Taiwo Opeyemi Aremu

26 Jul 2026

Social and Emotional Skills for Nurturing Parenting: A Qualitative Examination

PONE-D-25-63685R1

Dear Dr. Cuartas,

We’re pleased to inform you that your manuscript has been judged scientifically suitable for publication and will be formally accepted for publication once it meets all outstanding technical requirements.

Within one week, you’ll receive an e-mail detailing the required amendments. When these have been addressed, you’ll receive a formal acceptance letter and your manuscript will be scheduled for publication.

An invoice will be generated when your article is formally accepted. Please note, if your institution has a publishing partnership with PLOS and your article meets the relevant criteria, all or part of your publication costs will be covered. Please make sure your user information is up-to-date by logging into Editorial Manager at Editorial Manager® and clicking the ‘Update My Information' link at the top of the page. For questions related to billing, please contact billing support.

If your institution or institutions have a press office, please notify them about your upcoming paper to help maximize its impact. If they’ll be preparing press materials, please inform our press team as soon as possible -- no later than 48 hours after receiving the formal acceptance. Your manuscript will remain under strict press embargo until 2 pm Eastern Time on the date of publication. For more information, please contact onepress@plos.org.

Kind regards,

Taiwo Opeyemi Aremu, MD, MPH, PhD

Academic Editor

PLOS One

Additional Editor Comments (optional):

Reviewers' comments:

Reviewer's Responses to Questions

**Comments to the Author**

Reviewer #1: (No Response)

Reviewer #2: All comments have been addressed

**********

2. Is the manuscript technically sound, and do the data support the conclusions?

Reviewer #1: Yes

Reviewer #2: Yes

**********

3. Has the statistical analysis been performed appropriately and rigorously?

Reviewer #1: N/A

Reviewer #2: N/A

**********

4. Have the authors made all data underlying the findings in their manuscript fully available?

The PLOS Data policy

Reviewer #1: (No Response)

Reviewer #2: No

**********

5. Is the manuscript presented in an intelligible fashion and written in standard English?

Reviewer #1: Yes

Reviewer #2: Yes

**********

Reviewer #1: Thank you for responding to the review comments. While I think the results could still do with more contextualization and meaning making, the presentation has been improved sufficiently enough to align with the descriptive nature of this report. While most comments have been adequately responded to, I would highlight two for review:

1. Authors have used n to quantify qualitative findings. I would discourage the N as the focus of conveying an idea generated in the codes and themes. It does not add anything over simply saying most, a few participants (for example).

2. The framing of the research question is limited to the 'What' and does not convey a qualitative enquiry. I would still encourage the authors to review.

Overall, the paper is improved from the original submission.

Reviewer #2: Thank you for the efforts in improving this important piece of work!

I am pleased with the changes and revisions.

**********

what does this mean?). If published, this will include your full peer review and any attached files.

If you choose “no”, your identity will remain anonymous but your review may still be made public.

**Do you want your identity to be public for this peer review?**  For information about this choice, including consent withdrawal, please see our Privacy Policy

Reviewer #1: No

Reviewer #2: **Yes:** Eunice Lobo

**********

# Editor report: Acceptance letter

**DOI:** 10.1371/journal.pone.0355716.r004

**Authors:** Taiwo Opeyemi Aremu

PONE-D-25-63685R1

PLOS One

Dear Dr. Cuartas,

I'm pleased to inform you that your manuscript has been deemed suitable for publication in PLOS One. Congratulations! Your manuscript is now being handed over to our production team.

At this stage, our production department will prepare your paper for publication. This includes ensuring the following:

* All references, tables, and figures are properly cited

* All relevant supporting information is included in the manuscript submission,

* There are no issues that prevent the paper from being properly typeset

You will receive further instructions from the production team, including instructions on how to review your proof when it is ready. Please keep in mind that we are working through a large volume of accepted articles, so please give us a few days to review your paper and let you know the next and final steps.

Lastly, if your institution or institutions have a press office, please let them know about your upcoming paper now to help maximize its impact. If they'll be preparing press materials, please inform our press team within the next 48 hours. Your manuscript will remain under strict press embargo until 2 pm Eastern Time on the date of publication. For more information, please contact onepress@plos.org.

You will receive an invoice from PLOS for your publication fee after your manuscript has reached the completed accept phase. If you receive an email requesting payment before acceptance or for any other service, this may be a phishing scheme. Learn how to identify phishing emails and protect your accounts at https://explore.plos.org/phishing.

If we can help with anything else, please email us at customercare@plos.org.

Thank you for submitting your work to PLOS ONE and supporting open access.

Kind regards,

PLOS ONE Editorial Office Staff

on behalf of

Dr. Taiwo Opeyemi Aremu

Academic Editor

PLOS One
